# hnRNPU-mediated pathogenic alternative splicing drives gastric cancer progression

**DOI:** 10.1186/s13046-024-03264-9

**Published:** 2025-01-07

**Authors:** Guoguo Jin, Yanming Song, Shaobo Fang, Mingyang Yan, Zhaojie Yang, Yang Shao, Kexin Zhao, Meng Liu, Zhenwei Wang, Zhiping Guo, Zigang Dong

**Affiliations:** 1 Henan Key Laboratory of Chronic Disease Management, Fuwai Central China Cardiovascular Hospital, Zhengzhou, 450000 China; 2 Department of Pathophysiology, School of Basic Medical Sciences, Zhengzhou University, Zhengzhou, Henan China; 3 China-US (Henan) Hormel Cancer Institute, No. 127, Dongming Road, Jinshui District, Zhengzhou, Henan China; 4 Department of Medical Imaging, Zhengzhou University People’s Hospital& Henan Provincial People’s Hospital, Zhengzhou, 450000 China; 5 Central China Subcenter of National Center for Cardiovascular Diseases, Henan Cardiovascular Disease Center, Fuwai Central-China Cardiovascular Hospital, Central China Fuwai Hospital of Zhengzhou University, Zhengzhou, 450046 China; 6 Tianjian Laboratory of Advanced Biomedical Sciences, Institute of Advanced Biomedical Sciences, Zhengzhou University, Zhengzhou, Henan China; 7 Laboratory of Bone Tumor, Luoyang Orthopedic Hospital of Henan Province (Orthopedic Hospital of Henan Province), Zhengzhou, 450000 China

**Keywords:** Gastric cancer, hnRNPU, FTO, Alternative splicing

## Abstract

**Background:**

Alternative splicing (AS) is a process that facilitates the differential inclusion of exonic sequences from precursor messenger RNAs, significantly enhancing the diversity of the transcriptome and proteome. In cancer, pathogenic AS events are closely related to cancer progression. This study aims to investigate the role and regulatory mechanisms of AS in gastric cancer (GC).

**Methods:**

We analyzed AS events in various tumor samples and identified hnRNPU as a key splicing factor in GC. The effects of hnRNPU on cancer progression were assessed through in vitro and in vivo experiments. Gene knockout models and the FTO inhibitor (meclofenamic acid) were used to validate the interaction between hnRNPU and FTO and their impact on AS.

**Results:**

We found that hnRNPU serves as a key splicing factor in GC, and its high expression is associated with poor clinical prognosis. Genetic depletion of hnRNPU significantly reduced GC progression. Mechanistically, the m^6^A demethylase FTO interacts with hnRNPU transcripts, decreasing the m^6^A modification levels of hnRNPU, which leads to exon 14 skipping of the MET gene, thereby promoting GC progression. The FTO inhibitor meclofenamic acid effectively inhibited GC cell growth both in vitro and in vivo.

**Conclusion:**

The FTO/hnRNPU axis induces aberrant exon skipping of MET, thereby promoting GC cell growth. Targeting the FTO/hnRNPU axis may interfere with abnormal AS events and provide a potential diagnostic and therapeutic strategy for GC.

**Graphical Abstract:**

Schematic model for the findings of this work: Aberrant hnRNPU in GC binds FTO. The m^6^A-modified hnRNPU transcripts are recognized by m^6^A reader YTHDF3 and subsequently demethylated by FTO. This demethylation enhances the stability of hnRNPU mRNA, consequently promoting alternative splicing of MET. The altered splicing pattern ultimately contributes to the proliferation of GC cells.

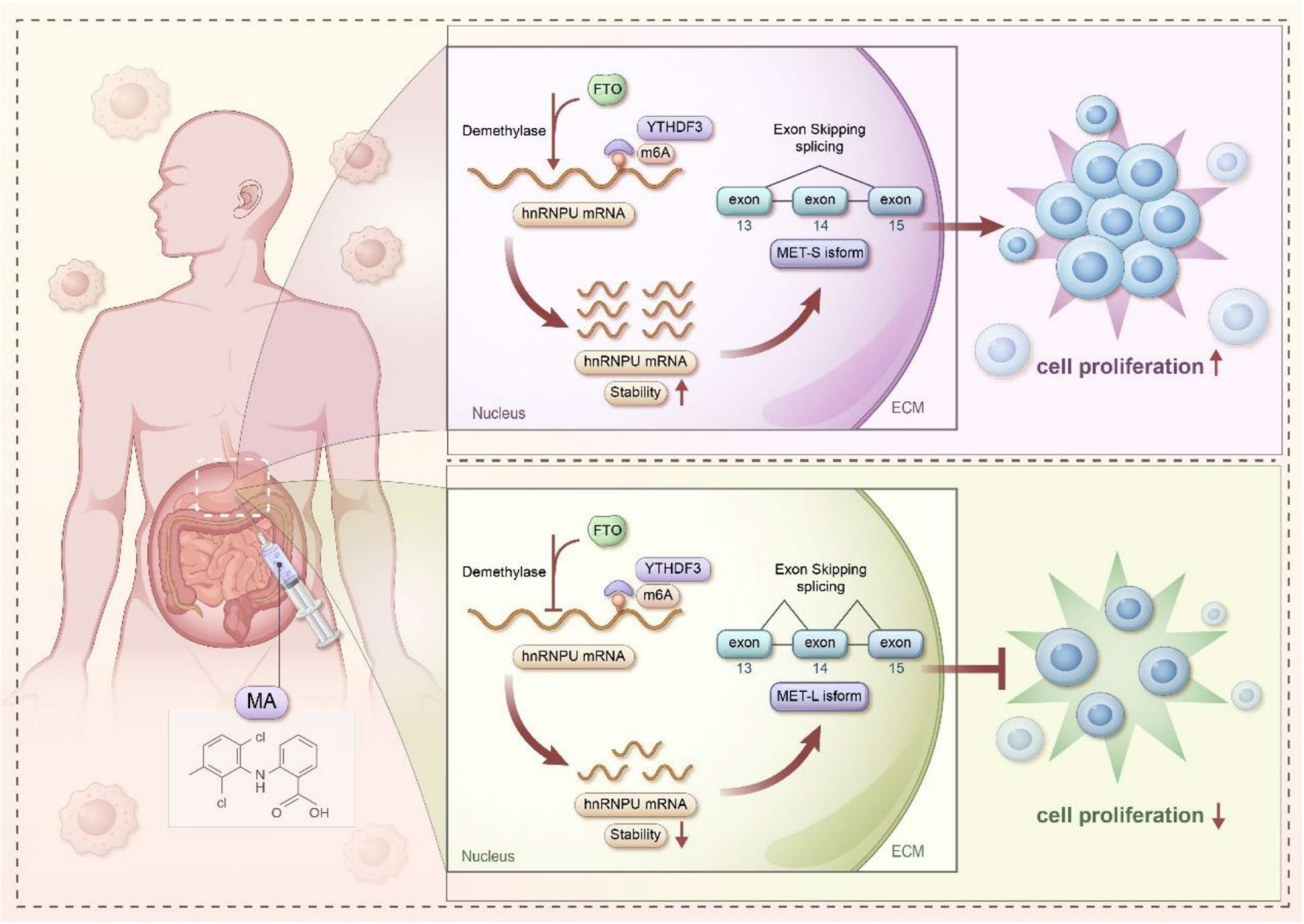

**Supplementary Information:**

The online version contains supplementary material available at 10.1186/s13046-024-03264-9.

## Background

Gastric cancer (GC), also known as stomach cancer, ranks as the fifth most prevalent malignancy worldwide and the third leading cause of cancer-related mortality globally [1, 2]. Despite the availability of several efficacious therapeutic interventions, the overall survival rates remain dismally poor [3, 4], highlighting an urgent need to understand the mechanisms governing the pathogenesis and progression of GC to develop more effective therapeutic strategies.

Alternative splicing (AS) is an important posttranscriptional regulation mechanism essential for cellular function [5, 6]. Those splicing events that contribute to disease progression can be classified as pathological alternative splicing [7]. Indeed, tumor tissues frequently exhibit extensive aberrations in splicing patterns compared to the adjacent tissues [8–10]. The heterogeneous nuclear ribonucleoproteins (hnRNPs) are a family of RNA-binding proteins (RBPs) that regulate AS as trans-acting factors and are implicated in tumor progression [11]. While previous studies have shown the role of specific hnRNPs, such as hnRNPU, in other cancers [12–14], the functional impact of hnRNPU in GC remain unexplored, making it a potential target for AS regulation in this disease. N^6^-methyladenosine(m^6^A) has been found to play an important role in the progression of human malignant tumors [15, 16]. Abnormal m^6^A modification is found in various tumors and is associated with tumor progression, metastasis, drug resistance, and prognosis [17–20]. Recent studies revealed the regulatory influence of m^6^A modifications on AS [21–23]. Fat mass and obesity-associated protein (FTO) was initially characterized as a gene implicated in obesity and energy metabolism and was then identified as the RNA m^6^A demethylase [24]. Previous studies indicate that FTO is highly expressed in many tumors and exhibits a critical promoting role in tumors through m^6^A modification-dependent mechanisms [25]. FTO is involved in cell proliferation, apoptosis, cell cycle, migration, invasion, and drug resistance [24]. FTO was found to play oncogenic roles in colorectal cancer [26], breast cancer [27], cervical cancer [28] and melanoma [29] though its post translation modification. However, the role of FTO in GC as an m^6^A demethylase remains poorly elucidated.

In this study, we identified an alternative splicing signature associated with the acquisition of malignant phenotypes in various types of cancer, and further elucidated hnRNPU as a key splicing factor dysregulated in GC. Remarkably, hnRNPU was found to be upregulated in GC tissues, correlating with poor prognosis. Both in vitro and in vivo experimental evidence demonstrated the oncogenic function of hnRNPU in promoting GC cell proliferation. Mechanistically, we illustrated that the m^6^A demethylase FTO binds to hnRNPU mRNA, thereby demethylating and reducing the m^6^A modification levels of hnRNPU, which consequently promoted the skipping of MET^exon 14^. while aberrant MET^exon14^ skipping promotes proliferation of GC cells. Collectively, our findings underscore the potential prognostic value and therapeutic implications of targeting this pathway in GC patients.

## Methods

### Cell culture and transfection

GC cell lines (HGC27, N87 and AGS) and HEK293T cells were procured from the American Type Culture Collection (ATCC; Manassas, VA). To ensure the accuracy and reliability of our experiments, we have conducted rigorous genetic authentication and mycoplasma contamination testing on all cell lines used. All cell lines have been confirmed to be free of genetic contamination, and no mycoplasma contamination was detected. The GC cell lines, HGC27, N87 and AGS, were propagated in RPMI 1640 medium supplemented with 10% heat-inactivated fetal bovine serum (FBS) and 1% penicillin/streptomycin antibiotic cocktail. In contrast, HEK293T cells were maintained in Dulbecco's modified Eagle's medium (DMEM) supplemented with 10% FBS. Cell culture conditions were maintained at 37 °C in a humidified incubator with a 5% CO_2_ atmosphere. For the transfection, shRNA or plasmids were transfected into cells using Lipofectamine 2000 reagent (Invitrogen Life Technol- ogy, USA) according to the manufacturer’s instructions and Simple-Fect Reagent (Signaling Dawn Biotech, Wuhan, Hubei, China) for HEK293T cells, according to the manufacturer’s instructions.

### Plasmid construction

Eukaryotic expression constructs, including pcDNA3.1-hnRNPU, pcDNA3.1-FTO, Flag-hnRNPU and Flag-FTO were obtained from Youbao Biotechnology Company (Changsha, China). Packaging vectors (PLKO.1, pMD2G, and psPAX2) were from Addgene Inc (Cambridge, MA, USA). The mutant plasmid of hnRNPU was generated using QuickMutation mutant kit (Beyotime, D0206S, Shanghai, China) and cloned into the pcDNA3.1-HA vector.

### Antibodies and reagents

Antibodies against the following proteins were used for Western blot: FTO (1:1000, proteintech; 27,226–1-AP), hnRNPU (proteintech; 14,599–1-AP) and (Cell signaling technology; 34095 T), MET (1:1000, proteintech; 25,869–1-AP), GAPDH (1:1000, ZSGB-BIO; TA-08), YTHDF3 (1:1000, proteintech; 25,537–1-AP), Flag-tag (1:1000, AFfinit; T0003).

### Cell proliferation and anchorage-independent growth assays

Cell proliferation was measured using the MTT assay. Cells were seeded in 96-well plates at a density of 1 × 10^3^ cells per well and absorbance was measured at 0, 24, 48, 72 and 96 h after seeding the cells. The colony formation capacity of GC cell lines was examined by an anchorage- independent growth assay. A total of 8,000 GC cells were suspended in triplicate in 1 mL of complete growth medium supplemented with 2 mM L-glutamine, 5 μg/mL gentamicin, and 0.3% low-melting-point agarose to form the top agar layer. The base layer consisted of 2 mL of 0.5% agarose in six-well plates. After an incubation period of 7–21 days, colonies were visualized and quantified using brightfield microscopy. Image-Pro Plus software (v.6.0) (Media Cybernetics, Rockville, MD) was employed for automated colony counting.

### Colony formation assay

GC cells were seeded into 6-well plates at a density of 500 cells/well and then were cultured for 14 days. The colonies were washed with 1X PBS and stained with 0.1% crystal violet. The colonies were counted under the microscope.

### Western blot

Cell pellets were resuspended in RIPA lysis buffer (50 mM Tris–HCl pH 7.4, 1 mM EDTA, 0.25% sodium deoxycholate, 1% Nonidet P-40, 150 mM NaCl, 0.1% SDS) and incubated on ice for 30 min to facilitate protein extraction. Following centrifugation at 12,000 × g for 10 min at 4 °C, the soluble protein fraction was collected from the supernatant. Protein quantification was performed using the BCA assay kit (Solarbio, Beijing, China, Cat#PC0020). Subsequently, 20–50 μg of total protein was denatured, resolved by SDS-PAGE, and transferred onto PVDF membranes. Membranes were blocked for 1 h at room temperature (RT) and then incubated with the appropriate primary antibody overnight at 4 °C, followed by incubation with a horseradish peroxidase (HRP)-conjugated secondary antibody for 2 h at RT. Protein bands were visualized using an enhanced chemiluminescence (ECL) detection reagent and captured with the Amersham Imager 800 (GE Healthcare Life Sciences, Pittsburgh, PA, USA).

### Immunohistochemistry assay

Human GC tissue microarray was procured from Shanghai Xinchao Biotechnology Company (Shanghai, China). All specimens underwent deparaffinization and rehydration processes. Antigen retrieval was subsequently performed by immersing the slides in boiling sodium citrate buffer (10 mmol/L, pH 6.0) for 90 s. Endogenous peroxidase activity was quenched by incubating the slides with 3% hydrogen peroxide for 10 min. Non-specific binding was blocked by treating the slides with 10% goat serum albumin for 30 min at RT. Slides were then incubated with specific primary antibodies overnight at 4 °C in a humidified chamber, followed by incubation with the corresponding secondary antibody for 30 min at RT. Subsequently, the slides were stained using 3,3'-diaminobenzidine and counterstained with hematoxylin. Immunohistochemistry (IHC) staining was quantified by calculating the percentage of positively stained cells using the Image-Pro Plus software (v.6.0) program (Media Cybernetics, Rockville, MD, USA).

### mRNA stability assay

The stability of hnRNPU mRNA was evaluated using the transcriptional inhibition assay. Cells were treated with actinomycin D (ActD; Sigma, A9415, USA) at a final concentration of 5 µg/mL to inhibit de novo transcription. At specific time points (0, 3 and 6 h) following ActD treatment, total RNA was extracted from the cells using a appropriate method (FastPure Cell/Tissue Total RNA Isolation, Vazyme). The abundance of hnRNPU mRNA at each time point was quantified by reverse transcription-quantitative real-time PCR (qPCR) using gene-specific primers. The mRNA decay kinetics were analyzed by plotting the relative hnRNPU mRNA levels (normalized to a stable reference gene) against the time after ActD treatment. The half-life (t1/2) of hnRNPU mRNA was calculated from the slope of the semi-log linear regression line, assuming first-order decay kinetics.

### RNA immunoprecipitation (RIP)

Immunoprecipitation targeting FTO and hnRNPU mRNAs was performed utilizing the RNA Immunoprecipitation (RIP) kit (Catalog No. KT-102–1, SaiCheng Bio, China) in accordance with the manufacturer's instructions. Total RNA was extracted from the cells using the FastPure Cell/Tissue Total RNA Isolation KIT (Vazyme, China). The extracted RNA was subsequently reverse-transcribed into complementary DNA (cDNA) using the HiScript III RT SuperMix for qPCR (Vazyme, China), following the manufacturer's protocol. Quantitative reverse transcription-polymerase chain reaction (qPCR) was conducted using the Taq Pro Universal SYBR qPCR Master Mix (Vazyme, China), and the analysis was performed on a 7500 Fast Real-Time PCR system (ThermoFisher Scientific, USA). The relative enrichment of RNA was normalized to the input.

### m^6^A-RNA immunoprecipitation assay (MeRIP-qPCR)

The m^6^A modification levels in mRNA were assessed using the methylated RNA immunoprecipitation (MeRIP) technique coupled with quantitative real-time PCR (qPCR). MeRIP was performed using the Magna MeRIP™ m^6^A Kit (Merck, 17–10,499, Germany) according to the manufacturer's instructions. Briefly, an anti-m^6^A antibody was first immobilized onto magnetic beads. Total RNA (100 µg) was then incubated with the antibody-bead complex in an RNase-inhibiting immunoprecipitation buffer overnight at 4 °C to allow binding of m^6^A-containing transcripts. After stringent washes, the m^6^A-enriched RNA fraction was eluted from the beads by proteinase K digestion and recovered by ethanol precipitation. The abundance of specific mRNA transcripts in the m^6^A-enriched and input RNA fractions was quantified by qPCR using gene-specific primers. The enrichment of m^6^A modification for each transcript was determined by normalizing the qPCR signal from the m^6^A-enriched fraction to that of the input RNA.

### RNA pull-down assay

The biotinylation of hnRNPU was carried out using the Pierce™ RNA 3' End Desthiobiotinylation Kit (Thermo Scientific, USA). RNA pulldown assays were performed employing the Pierce™ Magnetic RNA–Protein Pull-Down Kit (Thermo Scientific, USA) in accordance with the manufacturer's protocol. The immunoprecipitated protein was quantified and analyzed by Western blot assay.

### N6-methyladenosine (m^6^A) RNA methylation assay

Cells were rinsed three times with 1X PBS solution, and subsequently, total RNA was extracted from the cells using the FastPure Cell/Tissue Total RNA Isolation KIT (Vazyme, China). Quantification of methylated RNA was performed using the EpiQuik™ m^6^A RNA Methylation Quantification Kit (EpiQuik; P-9005–96) in accordance with the manufacturer's instructions, and the methylation levels were further analyzed using a microplate reader.

### Alternative splicing measured

A total RNA extract of GC cells was prepared using TRIzol reagent (15,596,026, Invitrogen, USA). The reverse transcription of RNA was performed using the HiScript® II Q Select RT SuperMix for qPCR (R233-01, Vazyme, China) as the manufacturer's instructions. The cDNA was used for qualitative real-time PCR using the Hieff® qPCR SYBR Green Master Mix (11202ES03, YEASEN, China).Percentspliced in index (PSI) was calculated using the formula:PSI = MET-S/(MET-L + MET-S).

The usage of primers for RT-qPCR were as follows:

- GAPDH forward, 5-TGCACCACCAACTGCTTAG-3, reverse, 5-GATGCAGGGATGATGTTC-3;
- MET-L(+ ex14) forward, 5-TCAACCGTCCTTGGAAAAGT-3, reverse,5-TGTGTACTCTTGCATCGTAGC-3;
- MET-S(-ex14), forward, 5-CAAATTAAAGATCAGTTTCC-3, reverse,5-AGCACTGAGGTCAATGTGGA-3;

### RNA-sequencing (RNA-seq) and data analysis

hnRNPU knockdown HGC27 cells (shhnRNPU) and wild-type HGC27 cells were selected for RNA sequencing analysis at Epibiotek Bio-tech Co., Ltd. (Guangzhou, China) using the Illumina high-throughput sequencing platform (NovaSeq 6000). Genome mapping of the reads for each sample was performed with a transcriptome built from GRCh37/hg19 using STAR version 2.7.6a. The reads were quantified by transcript abundances in transcripts per million mapped fragments (FPKM) using RSEM (version 1.2.15). The selection criteria for identifying differentially expressed RNAs were a *p*-value < 0.05 and a fold change > 1.

### Cell-derived xenograft (CDX) mouse model

Six- to eight-week-old NU/NU mice (Vital River Labs, Beijing, China) were randomly assigned into three experimental groups as follows: shNT (*n* = 7); shhnRNPU1 #1 (*n* = 7); shhnRNPU#2 (*n* = 7). Cells infected with the indicated lentivirus (HGC27: 1 × 10^7^ cells) were inoculated subcutaneously into the right flank of the mice. Tumor volumes were measured using a Vernier caliper and calculated according to the formula: V = (length) × (width) × (height) × 0.52. The mice were euthanized, and tumors were excised when the tumor volume reached 800 mm^3^ (cubic millimeters).

### Patient-derived xenograft (PDX) mouse model

Written informed consent was obtained from all patients whose cancer tissues were utilized in the study. Six- to eight-week-old female NOD/SCID mice (HFK Bio Science, Beijing, China) were used for in vivo experiments. Patient-derived tumor xenografts were established by implanting fresh surgical specimens from patients at the Affiliated Cancer Hospital of Zhengzhou University into the flanks of NOD/SCID mice. When tumors reached an average volume of approximately 150 mm^3^, mice were randomized into different treatment groups. Tumor volume (V) was calculated using the formula: V = (length × width^2^)/2, where length and width are the longest and shortest diameters, respectively.

### Generation of knockout mice

C57BL/6 J and Fto knockout mice were purchased from Cyagen Biosciences (China. Beijing).

Construction of FTO knockout mice using gRNAs, gRNA target sequence:

- gRNA1 (matching reverse strand of gene): TTTGGTAATCACGCATTGACAGG.
- gRNA2 (matching reverse strand of gene): CTTTCTCGGCAAACTAATCGTGG.

Validation of knockout results using PCR.

PCR Screening.

PCR Primers (Annealing Temperature 60.0 ºC):

- Forward primer (F1): 5’-GGGCAGTTACTCTCCTTACTCAG-3’.
- Reverse primer (R1): 5’-CGGTTTGCACAGGAAGTTCTAATAC-3’.
- Targeted allele: 729 bp Wildtype allele: 2770 bp.
- PCR Results: Animals 6, 7 and 8 were identified positive by PCR screening.

### N-methyl-N-nitrosourea (MNU) induced tumor in wild-type and Fto-knockout mice

Genomic DNA was extracted from mouse tail snips using a commercial genomic DNA extraction kit (TIANamp Genomic DNA Kit, TIANGEN). Genotyping of Fto knockout (*Fto*-cKO) mice was performed by polymerase chain reaction (PCR) amplification of the targeted region, followed by agarose gel electrophoresis to confirm the presence of the desired gene modification. Age-matched wild-type (WT) and *Fto*-cKO littermates were randomly assigned to either the control (water) or N-methyl-N-nitrosourea (MNU; Sigma, Cat# N4766) treatment group. MNU was administered in drinking water at a concentration of 120 ppm for 12 weeks. At the end of the treatment period, mice were euthanized, and stomachs were harvested. Each stomach was longitudinally sectioned; one half was fixed in formalin for histological analysis, including hematoxylin and eosin (H&E) staining and IHC, while the other half was snap-frozen for molecular analyses.

### Statistical analysis

All data are presented as mean ± standard deviation (SD) from at least three independent biological replicates, unless otherwise stated. Statistical analyses were performed using GraphPad Prism software (version 8.1.1, GraphPad Software, San Diego, CA, USA). For comparisons between two groups, the Student's t-test (two-tailed) was used. For multiple group comparisons, one-way analysis of variance (ANOVA) followed by Tukey's post hoc test was applied. Normality and homogeneity of variance were assessed using the Shapiro–Wilk and Levene's tests, respectively. In cases where the assumptions of parametric tests were violated, non-parametric alternatives (Mann–Whitney U test for two groups or Kruskal–Wallis test for multiple groups) were employed. Statistical significance was defined as *p* < 0.05 for all analyses.

## Results

### Identification of hnRNPU as a crucial regulator of AS events in GC

**Fig. 1:**
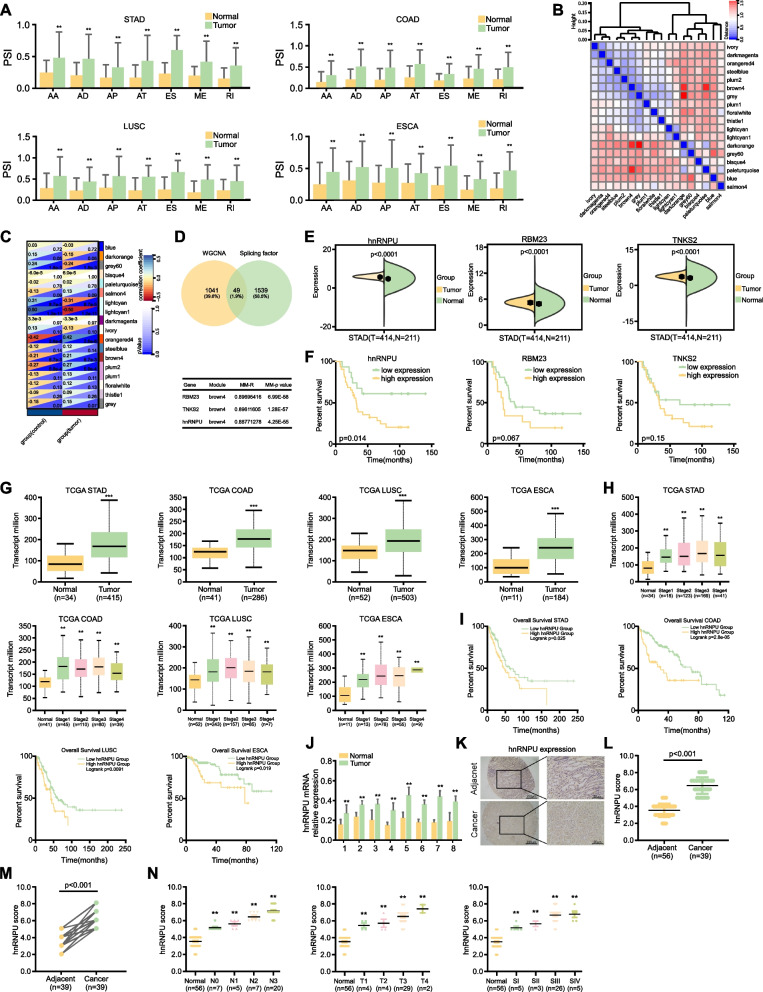
Identification of hnRNPU as a crucial regulator of AS events in GC. **A** The data comes from TCGA database, and the alternative splicing events is calculated between normal and tumor group. Statistical analysis was performed using the unpaired Student's t-test. **B** The data set GSE27342 is downloaded from GEO database, and then calculated and visualized based on the correlation between data matrices. The colors indicate the "distance" between genes and module eigengenes in WGCNA, which is a measure of the correlation of gene expression patterns. The intensity of blue indicates a stronger correlation, while the intensity of red signifies a weaker correlation. **C** Correlation between different modules and clinical traits. The colors in the upper left corner indicate the correlation coefficients (the bluer the color, the higher the correlation; the redder, the lower the correlation) and the colors in the lower right corner indicate the *p*-values (the bluer the color, the smaller the *p*-value). **D** Hub genes were identified by intersecting the results from WGCNA with known splicing factors. From this intersection, we selected the three genes exhibiting the most significant differential expression. **E** Analysis of expression levels of hnRNPU, RBM23, and TNKS2 in samples from TCGA datasets, comprising 414 primary GC tissues and 211 normal tissues. Different colors represent distinct sample groups. Statistical analysis was performed using the unpaired Student's t-test. **F** The relationship between the expression of hnRNPU, RBM23 and TNKS2 and the prognosis of GC. The *p* value was calculated using the log-rank test. **G** Differential expression of hnRNPU in gastric, colorectal, pulmonary, and esophageal carcinomas compared to adjacent normal tissues. Statistical analysis was performed using the unpaired Student's t-test. **H** hnRNPU protein levels across progressive stages of various cancer types. **I** Kaplan–Meier analysis of overall survival curve for GC (GC) patients with low and high hnRNPU levels, as characterized by TCGA database. The *p* value was calculated using the log-rank test.** J** Quantitative real-time PCR showing hnRNPU expression levels in GC tissues and adjacent tissues from 8 GC patients. Data are presented as mean ± SD. The *p*-value was determined using a two-tailed Student's t-test. **K** Representative immunohistochemistry images and evaluation of hnRNPU expression in a tissue microarray containing 56 GC tumors and 39 adjacent tissues. The *p*-value was determined using a two-tailed paired Student's t-test. **L**-**M** Quantitative analysis confirmed significantly higher hnRNPU protein level in GC tissue compared to the adjacent tissues. Statistical analysis was performed using the unpaired (**L**) paired (**M**) Student's t-test. **N** Stratifying by clinicopathologic characteristics, hnRNPU expression was dramatically increased with higher lymph node involvement, larger tumor size, and more advanced stage. Statistical analysis was performed using One-Way ANOVA. *p < 0.05, **p < 0.01, and ***p < 0.001 indicate significant differences between the groups

Alternative splicing is a crucial process that governs many aspects of cellular proliferation, survival, and differentiation [5, 6]. To elucidate the differential splicing events associated with neoplastic transformation, we performed a comprehensive analysis of The Cancer Genome Atlas (TCGA) database. Our investigation revealed a significant enrichment of alternative splicing events in various malignancies compared to normal tissue samples, including gastric, colorectal, lung, and esophageal carcinomas (data from TCGA database, accession number: phs000178) (Fig. 1A). Moreover, we downloaded the GSE27342 GC dataset from the GEO database and conducted an analysis using Weighted Gene Co-expression Network Analysis (WGCNA), resulting in the identification of 1041 hub genes (Fig. 1B, C). To identify the pivotal alternative splicing factor associated with GC, we downloaded splicing factor data from RBPDB (http://rbpdb.ccbr.utoronto.ca/proteins.php) and intersected with hub proteins to get 49 overlapping proteins. After ranking these proteins based on their associated *p*-values, the top three candidates were hnRNPU, RBM23 and TNKS2 (Fig. 1D). Ultimately, TCGA database was used to analyze the expression of hnRNPU, RBM23 and TNKS2 in GC, which showed that the expression levels of these three genes were elevated in GC (Fig. 1E). Subsequently, we used the data set GSE22377 from GEO database to investigate the potential association between the expression levels of hnRNPU, RBM23, and TNKS2 and the clinical outcomes in GC patients. The results showed that only hnRNPU was related to the prognosis of GC (Fig. 1F). These data showed that hnRNPU is the key splicing factor in GC. To elucidate the role of hnRNPU in cancer progression, we analyzed hnRNPU expression levels across various cancer types using TCGA database. Our analysis revealed significantly elevated hnRNPU expression in neoplastic tissues compared to adjacent normal tissues in gastric, colorectal, lung, and esophageal cancers (Fig. 1G). Furthermore, high hnRNPU expression was associated with advanced tumor stages (Fig. 1H) and correlated with poor prognosis (Fig. 1I). To verify the hnRNPU expression level in GC, q-PCR was used to detect the mRNA level of hnRNPU in tumor tissues and adjacent tissues. The results showed that the mRNA level of hnRNPU is upregulated in GC (Fig. 1J). To further confirm the role of hnRNPU in GC, we used a commercial GC tissue microarray containing 39 paired GC tissues and adjacent tissues for IHC. IHC results revealed that hnRNPU protein levels were higher in tumor versus unpaired normal GC tissues (Fig. 1K, L). hnRNPU was also elevated in paired GC tissues compared to normal tissues (Fig. 1M). Additionally, hnRNPU was upregulated in GC with greater lymph node involvement, larger tumor size, and more advanced clinical stage (Fig. 1N). Collectively, these findings indicate that hnRNPU is a crucial splicing factor implicated in aberrant AS events in GC, and highly expressed levels of hnRNPU are predictive of an unfavorable prognosis in GC patients. This suggests that hnRNPU could serve as an attractive therapeutic target for GC.

### hnRNPU promotes GC progression in vitro and in vivo

**Fig. 2:**
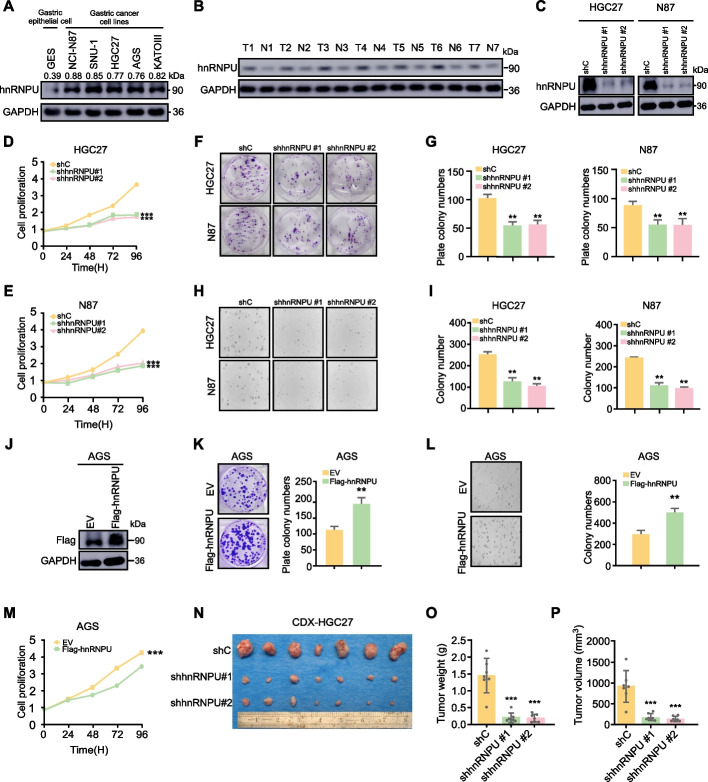
hnRNPU promotes GC progression in vitro and in vivo. **A**-**B** WB to detect the hnRNPU protein level in GC cell lines (NCI-N87, SNU-1, HGC27, AGS, KATOIII) and normal gastric epithelial cell (GES) or 7 paired patient gastric tumor and adjacent normal tissues. **C **The efficacy of two short hairpin RNAs (shRNAs), designated as #1 and #2, in knocking down hnRNPU expression was assessed by Western blot analysis in HGC27 and N87 GC cell lines. **D**-**E** MTT assay evaluating cellular proliferation of HGC27 and N87 cells following transduction with control (shC) or hnRNPU-targeted short hairpin RNA (shhnRNPU) vectors, maintained in RPMI-1640 medium supplemented with 10% heat-inactivated fetal bovine serum (FBS). Data represent the mean ± SD. Statistical significance was assessed via repeated measures two-way ANOVA. **F**-**I** Plate colony formation assay and anchorage-independent growth assays were used to assess the effect of knocking down hnRNPU on the proliferation ability of HGC27 and N87 cells, with representative images on the left and statistical graphs on the right. Data represent the mean ± SD. *p* value was calculated based on unpaired Student's t-test. **J** Western blot showed the overexpression of hnRNPU in AGS cells. **K**-**M** MTT assay showing the proliferation ability of AGS cells transfected with control (EV) vector or hnRNPU expression vector (hnRNPU) when cultured in 1640 medium supplemented with 10% FBS. Data are presented as mean ± SD. The *p*-value was determined using a unpaired Student's t-test. **N** To evaluate the effects of hnRNPU depletion on tumorigenicity, HGC27 GC cells with stable knockdown of hnRNPU expression via short hairpin RNAs (shRNAs) or non-targeting control shRNA were subcutaneously injected into athymic nude mice (*n* = 7 per group). Tumor weight (**O**) and tumor volume (**P**) measurements are presented and quantified in the right panel. Statistical analysis was performed using the unpaired Student's t-test. *p < 0.05, **p < 0.01, and ***p < 0.001 indicate significant differences between the groups

To explore the functional role of hnRNPU in GC, we first validate the expression levels of hnRNPU in GC. Initially, we checked the hnRNPU expression level in GC cells. Results showed that hnRNPU was highly expressed in GC compared with normal cells (Fig. 2A). Next, we collected 7 pairs of GC patient tissues from Affiliated Cancer Hospital of Zhengzhou University. Subsequent Western blot analyses revealed a significant upregulation of hnRNPU protein levels in the GC tissues compared with the adjacent normal tissue samples (Fig. 2B). To further investigate the role of hnRNPU in GC progression, we knock down hnRNPU in GC cell lines HGC27 and N87. Western blot analysis confirmed efficient depletion of hnRNPU protein levels (Fig. 2C). Knockdown of hnRNPU significantly decreased cell viability in HGC27 and N87 cells, as assessed by the MTT assay (Fig. 2D, E). Moreover, clone formation assays demonstrated a noticeable inhibition of colony formation upon hnRNPU knockdown (Fig. 2F, G). To further explore the impact of hnRNPU on gastric cell growth, we performed an anchorage-independent cell growth assay on hnRNPU knockdown cells, confirming that knocking down hnRNPU inhibited gastric cell growth (Fig. 2H, I). Conversely, we overexpressed hnRNPU in the GC cell line with the lowest hnRNPU expression (AGS) (Fig. 2J). The results showed that overexpression of hnRNPU promoted GC cells colony formation (Fig. 2K, L) and proliferation (Fig. 2M). To further verify the role of hnRNPU in vivo, we performed CDX model following hnRNPU knockdown (Fig. 2M). The results indicated that tumor size, weight, and tumor volume decreased upon knocking down hnRNPU (Fig. 2N, O, P). These results showed that hnRNPU promotes GC progression in vitro and vivo.

### Identification of FTO as an m^6^A eraser targeting hnRNPU

**Fig. 3:**
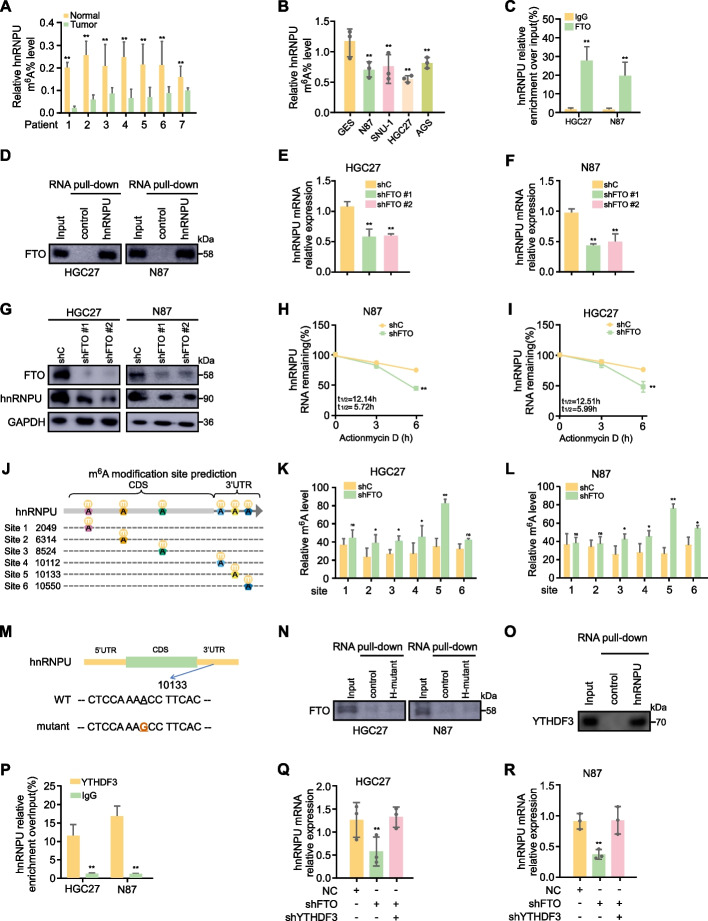
Identification of FTO as an m^6^A eraser targeting hnRNPU. **A**-**B** MeRIP-qPCR assay showing hnRNPU m^6^A modification levels in GC and adjacent tissues collected from 7 patients with GC. Data represent the mean ± SD. *p* value was calculated based on unpaired Student's t-test. **C** RIP assays were performed to evaluate the interaction between FTO and hnRNPU transcripts.* p* value was calculated based on unpaired Student's t-test. **D** RNA pull-down experiments were conducted to validate the interaction between FTO and hnRNPU transcripts. **E**–**G** qPCR and WB were employed to measure hnRNPU mRNA and protein levels following FTO knockdown in GC cell lines.* p* value was calculated based on unpaired Student's t-test. **H**-**I** Following FTO knockdown, the hnRNPU mRNA decay rate was analyzed using nonlinear regression analysis. *p* value was calculated based on repeated measures two-way ANOVA. **J** Potential m^6^A sites in hnRNPU transcripts. **K**-**L** MeRIP-qPCR assay showing hnRNPU m^6^A modification levels in HGC27 and N87 cells with control (shC) or hnRNPU knocdown vector (shhnRNPU) cultured in 1640 medium with 10% FBS. Data represent the mean ± SD. *p* value was calculated based on unpaired Student's t-test. **M** Schematic representation of the mutated m.^6^A modification site 5 in hnRNPU mRNA. **N.** RNA pull-down experiments to verify FTO interaction with hnRNPU transcripts following mutation of site 5. **O**-**P** RNA pull-down assay and RIP assays to evaluate the interaction between hnRNPU and YTHDF3. **Q**-**R** Quantitative real-time PCR showing hnRNPU expression levels in HGC27 and N87 cells with normal control (NC), FTO knockdown (shFTO) or YTHDF3 knockdown (shYTHDF3) cultured in 1640 medium with 10% FBS, Error bars denote mean ± SD. *p* value was calculated based on One-Way ANOVA. Significant differences between groups are indicated as *p < 0.05 and **p < 0.01

While previous studies have implicated m^6^A modifications as crucial regulators of GC progression, the relationship between aberrant alternative splicing and m^6^A modifications remains poorly elucidated [30, 31]. To verify whether the regulatory function of hnRNPU is modulated by m^6^A modifications, we collected tumor tissue and adjacent tissue specimens from 7 GC patients and assessed their respective m^6^A modification of hnRNPU levels. Our analysis revealed a significant reduction in the m^6^A modification levels of hnRNPU within the tumor tissues compared to the adjacent tissues (Fig. 3A). Subsequently, we detected m^6^A levels of hnRNPU in GC cell lines, and the results were consistent with our findings in tissue specimens (Fig. 3B). Based on these observations, we hypothesized that the m^6^A levels of hnRNPU might be regulated by m^6^A demethylases, specifically FTO and ALKBH5. To validate the interaction between hnRNPU and FTO or ALKBH5, we performed RNA immunoprecipitation (RIP) and RNA pull-down assays. The results demonstrated that only FTO, but not ALKBH5, could bind to hnRNPU mRNA (Figs. 3C, D, S1A). We then investigated the effects of FTO knockdown on hnRNPU expression. Following FTO depletion, we observed a significant decrease in both hnRNPU mRNA and protein levels (Fig. 3E-G). This was accompanied by an accelerated RNA decay rate compared to the control groups (Fig. 3H, I). Conversely, overexpression of FTO in AGS cells led to an increase in hnRNPU mRNA levels and a concomitant decrease in m^6^A levels of hnRNPU (Fig. S1B, C). This was associated with a decelerated RNA decay rate compared to the vector control groups (Fig. S1D). These results collectively support our hypothesis regarding the regulatory relationship between FTO and hnRNPU in GC cells.

Recent studies have elucidated the m^6^A-dependent roles of FTO in tumorigenesis and cancer progression across various malignancies [16, 18, 19]. To delineate the mechanism underlying FTO-mediated regulation of hnRNPU, we employed the SRAMP algorithm (http://www.cuilab.cn/sramp/) to predict potential m^6^A modification sites on hnRNPU transcripts, designating them as regions 1 through 6 (Fig. 3J). Following FTO knockdown in HGC27 and N87 cells, we observed the most significant increase in m^6^A levels within the site 5 region compared to control groups (Fig. 3K, L). To further validate these findings, we engineered a mutant plasmid construct for the site 5 region (Fig. 3M). Subsequent RNA pull-down assays confirmed that the site 5 region was indeed the most reliably modified region of hnRNPU transcripts (Fig. 3N). Collectively, these results demonstrate that FTO specifically demethylates hnRNPU m^6^A modifications within the site 5 region.

The prevalence and distribution of m^6^A modifications are determined by 'writers' and 'erasers', while 'readers' mediate the downstream effects of these modifications [32]. To date, several m^6^A reader proteins have been identified, primarily falling into two major families: the YT521-B homology (YTH) family, which includes YTHDF1, YTHDF2, and YTHDF3; and the insulin-like growth factor 2 mRNA-binding protein (IGF2BP) family, comprising IGF2BP1, IGF2BP2, and IGF2BP3 [33, 34]. To identify the specific m^6^A readers of hnRNPU transcripts, we conducted RNA pull-down and RNA immunoprecipitation (RIP) assays. Our findings revealed that YTHDF3, but not other readers, specifically interacted with hnRNPU transcripts in HGC27 and N87 cells (Fig. 3O, P, Fig. S1E-I). Knockdown of both FTO and YTHDF3 resulted in increased hnRNPU mRNA levels compared to FTO knockdown alone (Fig. 3Q, R). RIP assay results demonstrated that FTO knockdown enhanced the binding between hnRNPU transcripts and YTHDF3, while FTO overexpression attenuated this interaction (Fig. S1J-M). Together, these results suggest that hnRNPU is a downstream target of FTO, and that FTO modulates hnRNPU mRNA transcripts through the m^6^A reader YTHDF3.

### FTO overexpression correlates with poor prognosis

**Fig. 4:**
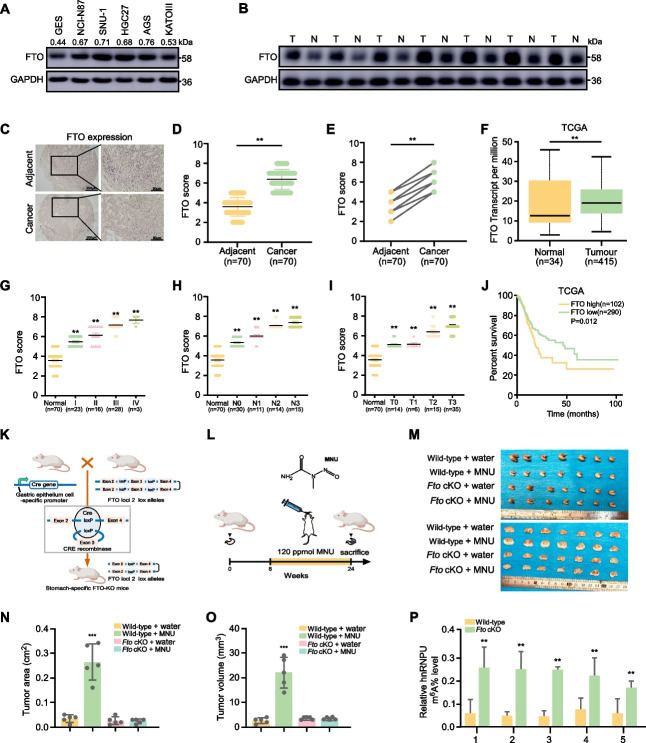
FTO overexpression correlates with poor prognosis. **A** FTO expression in normal gastric cell GES and GC cell lines NCI-N87, SNU-1, HGC27, AGS, and KATOIII. **B** Representative immunohistochemistry images and evaluation of FTO expression measured on a tissue microarray that contains 70 paired GC tumors andadjacent tissues. *p* value was calculated based on unpaired Student's t-test. **C**-**E** Quantitative analysis of FTO protein levels in GC tissue microarray based on IHC staining results. Statistical analysis was performed using the unpaired (D) paired (E) Student's t-test. **F** FTO expression in GC based on TCGA database analysis. *P* value was calculated based on unpaired Student's t-test. **G** FTO expression in patients with different clinical stages as determined by tissue microarray analysis. *p* value was calculated based on One-Way ANOVA. **H** Number of lymph node metastases (LNM) stratified by FTO expression levels.* p* value was calculated based on One-Way ANOVA. **I** FTO expression in relation to tumor size in tissue microarray analysis.* p* value was calculated based on One-Way ANOVA. **J** Kaplan–Meier analysis of survival curve of patients with GC with low (*n* = 290) and high (*n* = 103) hnRNPU levels characterized by TCGA database. *p* value was calculated based on log-rank test. **K** Schematic diagram illustrating the generation of stomach-specific conditional *Fto*-KO mice. **L** Schematic representation of the GC primary induction model. **M **Representative macroscopic images of stomachs from WT and *Fto*-KO mice. Quantitative analysis of tumor area (**N**) and tumor volume (**O**) in WT and FTO-KO mice with or without MNU treatment.* p* value was calculated based on One-Way ANOVA. **P** Relative hnRNPU m.^6^A levels in WT and *Fto*-KO groups. Statistical analysis was performed using the unpaired Student's t-test. Significant differences between groups are indicated as *p < 0.05 and **p < 0.01

To investigate the role of FTO in GC, we performed western blot experiments to examine the expression of FTO in normal gastric cell line GES and GC cell lines NCI-N87, SNU-1, HGC27, AGS, and KATOIII. Results demonstrated elevated FTO expression in GC cells compared to normal cells (Fig. 4A). We also examined FTO expression in 7 paired GC patient samples, including tumor and adjacent tissues, via Western blotting, revealing elevated expression in GC tissues (Fig. 4B). Subsequently, we performed IHC staining on a tissue microarray comprising tumor tissues (*n* = 70) and adjacent tissues (*n* = 70) to determine FTO protein levels. IHC analysis corroborated the upregulation of FTO in GC tissues compared to adjacent tissues (Fig. 4C-E). These findings were further validated using TCGA database (Fig. 4F). Elevated FTO expression was found to correlate with advanced clinical grade (Fig. 4G), lymph node metastasis (Fig. 4H), larger tumor size (Fig. 4I), and poor survival outcomes (Fig. 4J). To further verify the role of FTO in GC development, we generated *Fto* conditional knockout (*Fto*-cKO) and wild-type (WT) mice and induced gastric tumors using N-methyl-N-nitrosourea (MNU) (Fig. 4K, L). At 24 weeks post-induction, stomachs were harvested, revealing that *Fto*-cKO mice maintained a fuller stomach shape compared to the MNU-induced WT group (Fig. 4M). Following MNU induction, both tumor area and weight were significantly decreased in the *Fto*-cKO group compared to the WT group (Fig. 4N, O). Using this *Fto*-cKO mouse model, we assessed hnRNPU m^6^A levels after FTO knockout. Results indicated increased hnRNPU m^6^A levels in *Fto*-cKO mice compared to WT controls (Fig. 4P). Collectively, these findings demonstrate that FTO is highly expressed in GC and promotes GC progression both in vitro and in vivo.

### FTO promotes GC progression in m^6^A-dependent manner

**Fig. 5:**
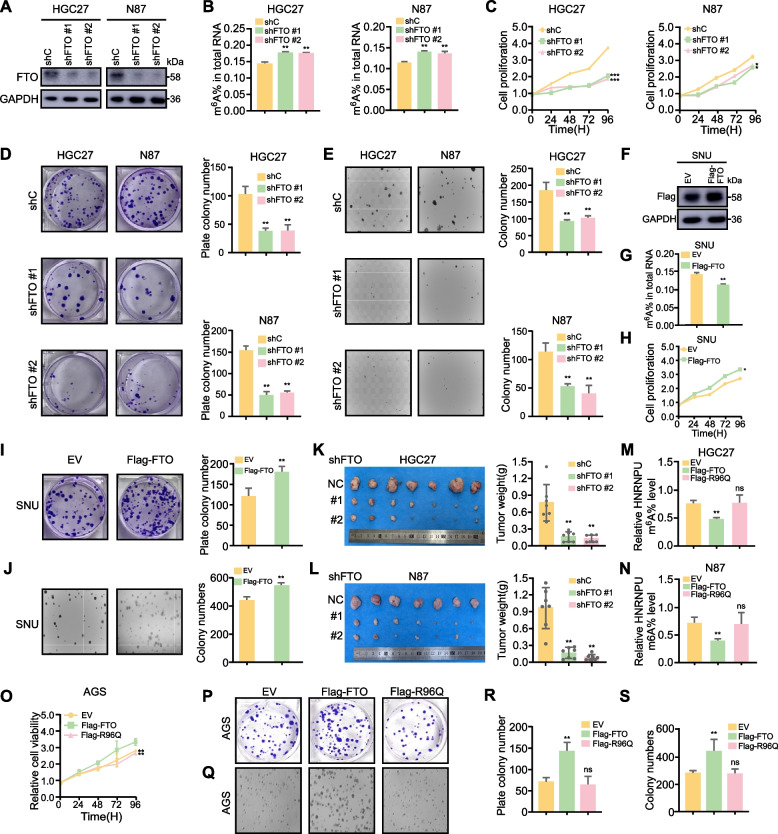
FTO promotes GC progression in m^6^A-dependent manner. **A** Establishment and validation of FTO knockdown and overexpression in cells, confirmed by Western blot analysis. **B **EpiQuik M^6^A RNA Methylation Quantification Kit assay showing total m^6^A modification levels in HGC27 and N87 cells with control (shC) or FTO knocdown vector (shFTO) cultured in 1640 medium with 10% FBS. Data represent the mean ± SD. *p* value was calculated based on One-Way ANOVA. **C**-**E** MTT, Plate colony formation and anchorage-independent growth assays showing proliferation ability of HGC27 and N87 cells with control (shC) or FTO knocdown vector (shFTO) cultured in 1640 medium with 10% FBS. Data represent the mean ± SD. *p* value was calculated based on one-Way ANOVA (D, E) and two-Way ANOVA (C). **F**-**I** MTT, Plate colony formation and anchorage-independent growth assays showing proliferation ability of SUN cell with control (EV) vector or FTO expression vector (FTO) cultured in 1640 medium with 10% FBS. Data represent the mean ± SD. *P* value was calculated based on two-tailed Student’s t test. **J** EpiQuik M^6^A RNA Methylation Quantification Kit assay showing total m^6^A modification levels of HGC27 and N87 cells with with control (EV) vector or FTO expression vector (FTO) cultured in 1640 medium with 10% FBS. Data represent the mean ± SD. Statistical analysis was performed using the unpaired Student's t-test. **K**, **L** Subcutaneous injection of HGC27 and N87 cells stably expressing shNT, shFTO#1, or shFTO#2 into the right flank of nude mice (*n* = 7 per group). Analysis of tumor weight in CDX mouse model in right panel. *p* value was calculated based on One-Way ANOVA. **M**,** N** Impact of FTO demethylase mutant R96Q on hnRNPU m.^6^A levels in HGC27 and N87 cells.* p* value was calculated based on One-Way ANOVA.** O** MTT assay showing proliferation ability of HGC27 and N87 cells with control (EV) vector, FTO expression vector (FTO) and FTO-R96Q mutant expression vector (R96Q) cultured in 1640 medium with 10% FBS. Data represent the mean ± SD. *p* value was calculated based on unpaired Student's t-test. **P**,** Q R**,** S** Quantitative analysis of plate crystal violet staining and soft agar colony formation assays**.** Statistical analysis was performed using one-Way ANOVA (R, S) and two-Way ANOVA (O). Significant differences between groups are indicated as *p < 0.05 and **p < 0.01

To further elucidate the effect of FTO on GC progression, we performed FTO knockdown in the GC cell lines HGC27 and N87. Western blot analysis confirmed successful FTO depletion (Fig. 5A). Given FTO's role as an m^6^A eraser, we assessed total RNA m^6^A levels following FTO knockdown, observing a significant increase (Fig. 5B). Furthermore, we assessed HGC27 and N87 cells proliferation and observed a significant suppression after FTO knockdown (Fig. 5C). Moreover, clone formation was inhibited following FTO knockdown (Fig. 5D). Additionally, we performed an anchorage independent cell growth assay to confirm the inhibitory effect after knocking down FTO (Fig. 5E). Conversely, FTO overexpression in the SNU cell line (Fig. 5F) led to decreased total RNA m^6^A levels (Fig. 5G) and enhanced cell proliferation and colony formation (Fig. 5H-J). To validate the role of FTO in vivo, we established CDX models using FTO-knockdown HGC27 and N87 cells. Results showed decreased tumor weight following FTO depletion (Fig. 5K, L). To investigate the role of the FTO-hnRNPU axis in GC progression, we overexpressed hnRNPU in FTO knockdown cell lines. Our results demonstrated that hnRNPU overexpression in FTO knockdown cells attenuated the inhibitory effect on cell proliferation and colony formation observed with FTO depletion alone (Fig. S2A-E).

In order to verify FTO promotes GC progression in m^6^A-dependent manner, we engineered an FTO mutant (R96Q) lacking m^6^A demethylation activity. GC cell growth was inhibited in FTO mutant groups (Fig. 5O). We also assessed hnRNPU m^6^A levels after FTO mutation. While FTO overexpression decreased hnRNPU m^6^A levels, the FTO mutant showed no change compared to the vector control (Fig. 5M, N). Colony formation assays revealed that the number of colonies in the FTO mutant group exhibited no significant difference when compared to the vector control group (Fig. 5P-S). Collectively, these results provide compelling evidence that FTO promotes GC progression in an m^6^A-dependent manner.

### hnRNPU controlled the exon skipping of MET in GC cells

**Fig. 6:**
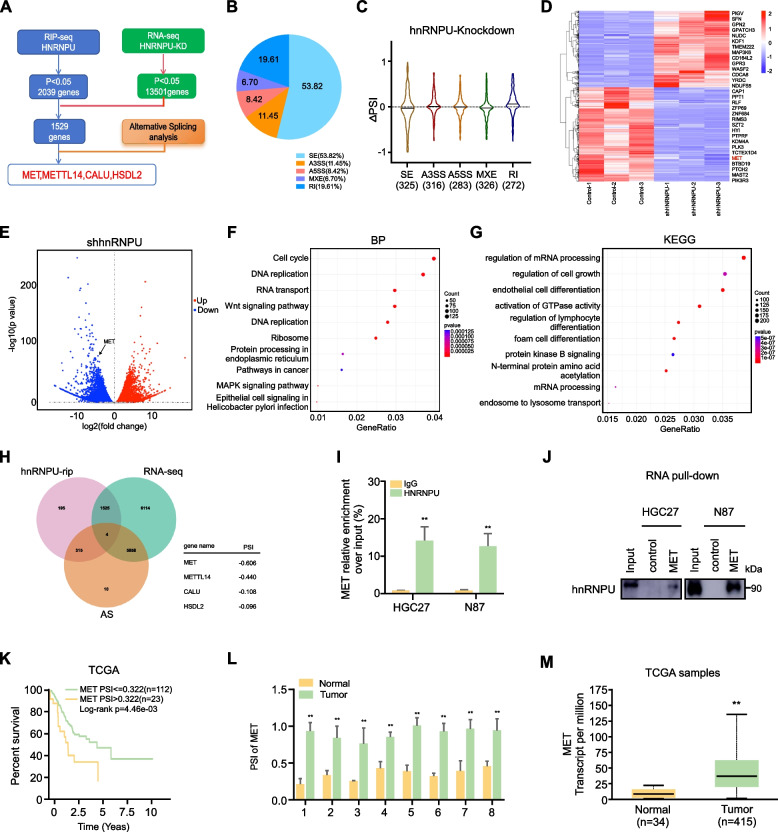
hnRNPU controlled the exon skipping of MET in GC cells. **A** Schematic workflow of RNA-seq and RIP-seq. **B** Distribution of different types of alternative splicing (AS) events. **C** Violin plots of changes of the significant percent splicing inclusion (ΔPSI) in the hnRNPU knockdown cells. **D** Heatmap visualization of gene expression changes following hnRNPU knockdown in HGC27 cells. **E** Volcano plot of gene changes after knockdown of hnRNPU **F**. Gene Ontology analysis of biological processes affected in hnRNPU-knockdown HGC27 cells. **G** KEGG pathway analysis in hnRNPU-knockdown HGC27 cells. **H** Venn diagram showing four genes (MET, METTL14, CALU, HSDL2) identified at the intersection of hnRNPU-RIP, RNA-seq, and AS datasets. **I**,**J** RIP and RNA pull-down assays confirming the interaction between hnRNPU and MET mRNA. **K** Kaplan–Meier analysis of survival curve of patients with GC with low MET PSI values (*n* = 112) and high MET PSI values (*n* = 23) characterized by TCGA database. *p* value was calculated based on log-rank test. **L** Quantitative real-time PCR showing MET PSI values in GC tissues and adjacent tissues from 8 GC patients. Data are presented as mean ± SD. The *p*-value was determined using an unpaired Student's t-test. **M** Analysis of the expression levels of MET between samples of GC and normal that contain 415 primary GC and 34 normal patients in TCGA datasets. Different colors refer to different samples. Significant differences between groups are indicated as *p < 0.05 and **p < 0.01

To gain further insight into the tumorigenic mechanisms of hnRNPU, we conducted high-throughput mRNA sequencing (RNA-seq) using hnRNPU knockdown cells and RNA immunoprecipitation sequencing (RIP-seq) using cell lines with high hnRNPU expression. The experimental workflow is depicted in Fig. 6A. Various types of AS events are illustrated in Fig. 6B. Upon hnRNPU knockdown, all types of AS events were affected, with skipped exon (SE) events being the most prevalent in HGC27 cells (Fig. 6C). Heatmap analysis revealed a significant decrease in MET mRNA levels after hnRNPU knockdown (Fig. 6D). Volcano plot analysis corroborated the alteration in MET mRNA levels afte hnRNPU depletion (Fig. 6E). In GC cell lines, biological processes related to DNA replication were frequently observed to be altered (Fig. 6F). KEGG pathway analysis of the RNA-seq data indicated that hnRNPU regulates cell growth pathways (Fig. 6G). Given the role of hnRNPU in regulating pre-mRNA alternative splicing, we hypothesized that hnRNPU-mediated cell growth in GC might occur through hnRNPU-dependent alternative splicing. To test this hypothesis, we analyzed the overlap between RIP-seq and RNA-seq data, identifying 1,529 common genes. Further analysis of AS events in GC revealed four genes (MET, METTL14, CALU, and HSDL2) with significantly altered Percent Spliced In (PSI) values (Fig. 6H). Based on the PSI values of the four candidates, we selected MET for further validation. RIP and RNA pull-down assays confirmed the interaction between hnRNPU and MET mRNA (Fig. 6I, J). MET, a well-known oncogene in various tumors, has been associated with tumor growth through alternative splicing. Kaplan–Meier analysis demonstrated that high PSI values for MET predict poor prognosis (Fig. 6K). We then examined the PSI of MET in 8 paired gastric patient tissue samples. Results showed that tumor tissues exhibited higher PSI values for MET compared to adjacent tissues (Fig. 6L). MET also highly expressed in gastric tumor tissues compared with normal tissues (Fig. 6M). Collectively, these data demonstrate that hnRNPU modulates alternative splicing in GCs and specifically regulates the exon skipping of MET.

### hnRNPU enhances proliferation in GC cells by promoting the skipping of exon 14 in the MET proto-oncogene transcript

**Fig. 7:**
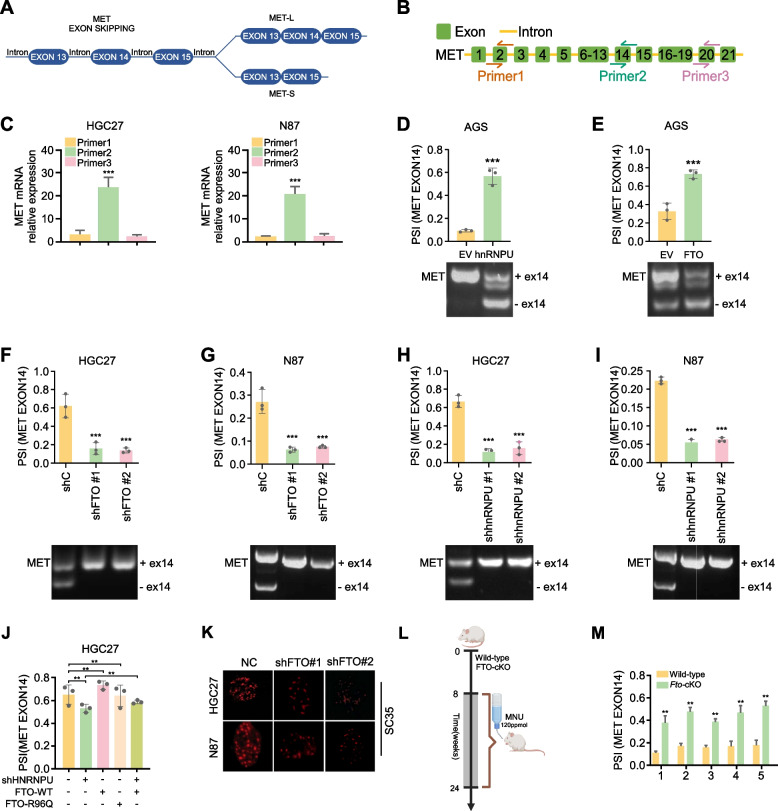
hnRNPU enhances proliferation in GC cells by promoting the skipping of exon 14 in the MET proto-oncogene transcript. **A** Schematic representation of alternative splicing patterns in MET. **B** Design of specific primers labeled as primer 1 to primer 3. **C** RIP assays to detect the specific interaction sites between hnRNPU and MET. Statistical analysis was performed using one-Way ANOVA **D**, **E**. Quantitative real-time PCR assay showing PSI of MET exon 14 skipping in AGS cell with with control (EV) vector, hnRNPU expression vector (hnRNPU) or FTO expression vector (FTO) cultured in 1640 medium with 10% FBS. Data represent the mean ± SD. *p* value was calculated based on unpaired Student's t-test **F-I**. Quantitative real-time PCR assay showing PSI of MET exon 14 skipping in HGC27 and N87 cells with control (shC), hnRNPU knocdown vector (shhnRNPU) or FTO knockdown vector (shFTO) in 1640 medium with 10% FBS. Data represent the mean ± SD. *p* value was calculated based on one-Way ANOVA. **J** Comparison of PSI values in hnRNPU knockdown cells, FTO wild-type (WT) cells, and FTO mutant cells.* p* value was calculated based on one-Way ANOVA. **K** Nuclear speckle assay to assess splicing activity following FTO knockdown. **L**. The schematic of MUN induced *Fto*-KO mouse model. **M** Analysis of MET exon 14 skipping PSI in WT and *Fto*-KO tumor tissues. The *p*-value was determined using an unpaired Student's t-test. Significant differences between groups are indicated as *p < 0.05 and **p < 0.01

AS events in MET play a crucial role in tumorigenesis [35, 36]. To explore the function of MET AS events in GC, we present a schematic of the alternative splicing patterns of MET in Fig. 7A. To identify precise interaction sites between hnRNPU and MET, we designed specific primers labeled as primer 1 to primer 3 (Fig. 7B). RIP assays revealed that hnRNPU predominantly binds to exon 14 in MET (Fig. 7C). We then elucidated the impact of FTO and hnRNPU on the AS event resulting in MET exon 14 skipping in GC cells. hnRNPU or FTO overexpression increased the PSI of MET exon 14 skipping (Fig. 7D, E). Knockdown of FTO or hnRNPU resulted in decreased PSI of MET exon 14 skipping (Fig. 7F-I). To validate the FTO-hnRNPU axis in regulating MET splicing, we overexpressed wild-type or mutant FTO in hnRNPU knockdown cell lines. Our results demonstrated that this overexpression restored the PSI value to levels comparable to those observed in control cells, in contrast to the altered PSI values seen in the FTO mutant and hnRNPU knockdown groups (Fig. 7J).

Nuclear speckles are implicated in the post-transcriptional regulation of gene expression, particularly in pre-mRNA splicing [37]. To elucidate the effect of FTO on splicing activity, we performed immunofluorescence assays on FTO-knockdown cells using anti-SC35, a well-established nuclear speckle marker. Our results demonstrated that FTO knockdown decreased spliceosome binding to pre-mRNA and resulted in lower splicing levels compared to FTO located farther from nuclear speckles (Fig. 7K). This observation suggests a potential role for FTO in modulating the spatial organization and efficiency of splicing machinery within the nucleus.

Using the FTO knockout mouse model, we observed decreased PSI of MET exon 14 skipping upon FTO deletion (Fig. 7L, M). To elucidate the function of MET AS events in GC progression, we first examined the expression levels of MET-L (+ exon 14) and MET-S (-exon 14) in GC cells. Results showed that MET-S was highly expressed in tumor cells (Fig. S3A, B). MTT and soft agar assays demonstrated that MET-S increased cell proliferation and colony formation (Fig. S3C-H). In rescue experiments, overexpression of MET-S, but not MET-L, restored cell proliferation and colony formation following hnRNPU knockdown (Fig. S3I, J). Collectively, these data demonstrate that hnRNPU-mediated exon 14 skipping in MET promotes cell growth in GC.

### Identification of GC prognosis-related AS events

**Fig. 8:**
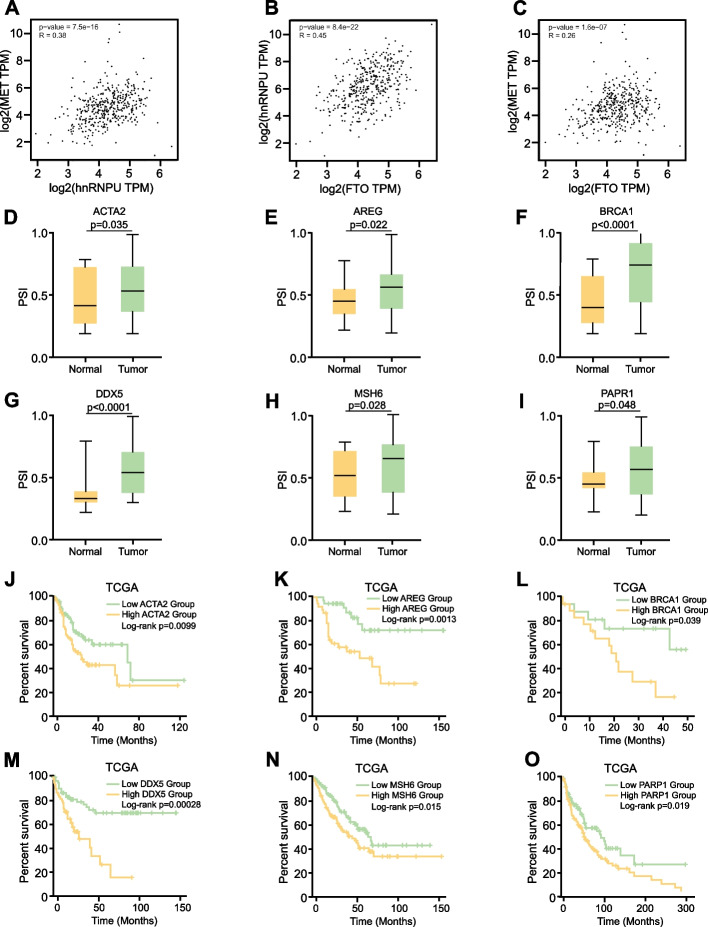
Identification of GC prognosis-related AS events. **A**-**C** Analysis of protein expression level correlations between hnRNPU and MET, FTO and hnRNPU, and FTO and MET using the Gene Expression Profiling Interactive Analysis (GEPIA) database. **D**-**I** Comparative analysis of PSI values for ACTA2, AREG, BRCA1, DDX5, MSH6, and PARP1 in tumor tissues versus adjacent normal tissues using TCGA database. **J**-**O** Evaluation of the relationship between PSI values of the aforementioned genes and clinical prognosis. *p* value was calculated based on log-rank test

Our previous findings demonstrated that the FTO-hnRNPU-MET axis plays a pivotal role in GC progression. To further elucidate the relationship among these proteins, we conducted an analysis using the GEPIA database. We observed positive correlations between hnRNPU and MET protein expression levels, FTO and hnRNPU protein expression levels, and FTO and MET protein expression levels (Fig. 8A-C). Consistent with our previous data, elevated expression of all three proteins was associated with poor prognosis. Our prior results established the crucial role of AS events in GC progression. To extend these findings, we analyzed the TCGA database, focusing on genes previously reported to predict clinical outcomes in GC [38]. Analysis of TCGA database (accession number: phs000178) revealed increased PSI values for ACTA2, AREG, BRCA1, DDX5, MSH6, and PARP1 in tumor tissues compared to adjacent tissues (Fig. 8D-I). Moreover, high PSI values for these genes were predictive of poor prognosis (Fig. 8J-O). Collectively, these data underscore the widespread occurrence of AS events in GC and suggest that ACTA2, AREG, BRCA1, DDX5, MSH6, and PARP1 could serve as potential biomarkers for GC prognosis and progression.

### Meclofenamic acid (MA), a selective inhibitor of FTO, attenuates GC progression in vitro and in vivo

**Fig. 9:**
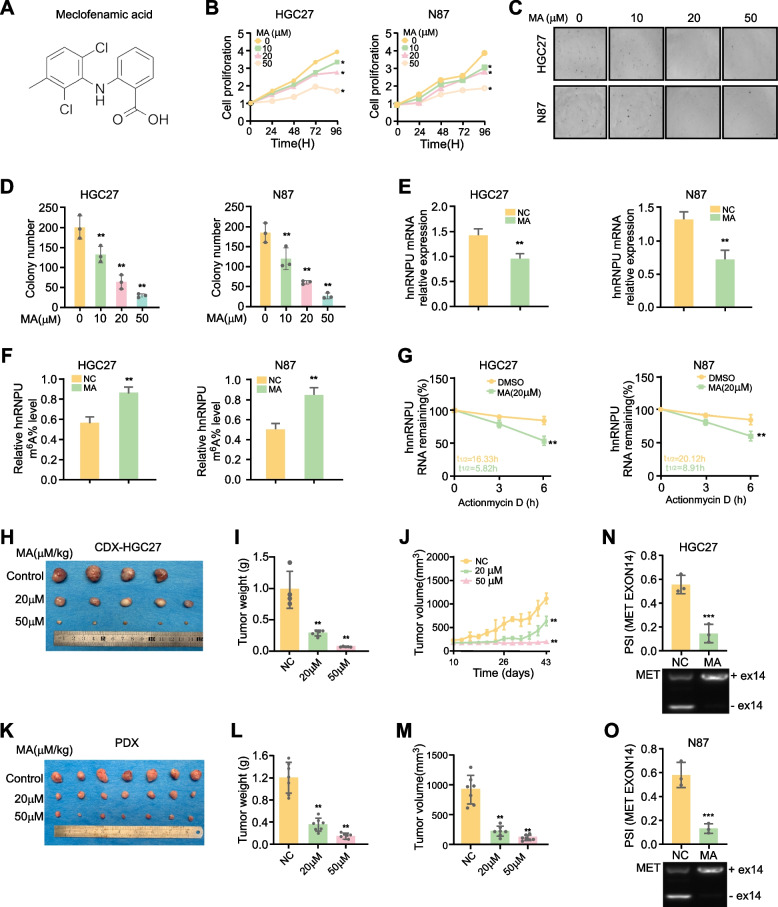
Meclofenamic acid (MA), a selective inhibitor of FTO, attenuates GC progression in vitro and in vivo. **A** The chemical structure of MA. **B** MTT assay showing proliferation ability of HGC27 and N87 cells with 0 μm,10 μm, 20 μm, 30 μm, 40 μm, 50 μm MA cultured in 1640 medium with 10% FBS. Data represent the mean ± SD. *p* value was calculated based on two-Way ANOVA. **C**,** D** Plate colony formation assay showing proliferation ability of HGC27 and N87 cells with 0 μm,10 μm, 20 μm, 50 μm MA cultured in 1640 medium with 10% FBS. Data represent the mean ± SD. *p* value was calculated based on one-Way ANOVA **E**. Relative hnRNPU mRNA levels following MA treatment, measured by qPCR. The *p*-value was determined using an unpaired Student's t-test.** F** hnRNPU m.^6^A level was checked after treatment with MA. The *p*-value was determined using an unpaired Student's t-test. **G** RNA decay rates in response to MA treatment. *p* value was calculated based on two-Way ANOVA. **H**-**J** MA effect on GC using HGC27 CDX model, with representative images, tumor weight and tumor volume were quantification. **K**-**M** PDX model assessment of MA effect on GC, including representative images, and tumor weight analysis. *p* value was calculated based on two-Way ANOVA. **N**, **O** Effect of FTO inhibitor on the PSI of MET exon 14 skipping. The *p*-value was determined using an unpaired Student's t-test. **P < 0.01, ***P < 0.001

In this study, we demonstrated that FTO plays a crucial role in GC progression, thereby establishing FTO targeting as a potentially effective strategy for GC therapy. To date, there are no Food and Drug Administration (FDA)-approved FTO inhibitors for clinical use as therapeutic agents. Meclofenamic acid (MA), a non-steroidal anti-inflammatory drug, has been shown through mechanistic studies to compete with FTO for binding to m^6^A-containing nucleic acids. Therefore, we sought to investigate the effects of MA on GC cell growth. The chemical structure of MA was shown in Fig. 9A. MTT and soft agar assays revealed that MA inhibited GC cell growth in a dose-dependent manner (Fig. 9B-D). Following MA treatment, we observed a decrease in hnRNPU mRNA levels, an increase in hnRNPU m^6^A levels (Fig. 9E, F), and a reduced RNA decay rate (Fig. 9G). To further evaluate the efficacy of MA, we utilized both GC PDX and CDX mouse models. MA treatment significantly reduced tumor weight and volume in both models (Fig. 9H-M). Additionally, treatment with the FTO inhibitor significantly decreased the percent spliced in (PSI) of MET exon 14 skipping (Fig. 9N, O). In conclusion, these data collectively demonstrate that MA exhibits antiproliferative effects on GC cell lines through its targeted inhibition of FTO. Furthermore, these findings suggest that MA may have potential as a therapeutic agent for GC treatment.

## Discussion

Alternative splicing, an essential step in gene expression, has been increasingly implicated in a growing number of human diseases, particularly cancer [39]. Our analysis of TCGA database revealed a significant enrichment of alternative splicing events in GC samples compared to normal tissues, suggesting that aberrant alternative splicing is a major contributor to GC progression. In this study, we identified hnRNPU as a key modulator of alternative splicing in GC progression. Previous studies on hnRNPU in cancer have primarily focused on its proliferative properties in breast cancer [12], hepatocellular carcinoma [14], multiple myeloma [13], and bladder cancer [40]. However, the function and tumorigenic role of hnRNPU in GC have been largely unexplored, which prompted our investigation. Our study demonstrated that hnRNPU promotes the proliferation of GC cells both in vitro and in vivo. This finding was corroborated by patient data showing elevated expression of hnRNPU in cancer tissues compared to adjacent tissues. Moreover, high hnRNPU expression was significantly associated with poor prognosis, consistent with observations in other human cancers. These results collectively suggest that hnRNPU functions as an oncogene in GC.

hnRNPU has been characterized as an RNA-binding protein and previously demonstrated to function as a global splicing regulator [41]. Han et al. reported that hnRNPU interacts with DDX5 to modulate alternative splicing and transcription [12]. In our study, we found that hnRNPU binds to MET mRNA, as verified by RIP and RNA pull-down assays. RNA-seq and RIP-seq analyses suggested that hnRNPU modulates alternative splicing and transcription by interacting with MET and promoting MET exon 14 skipping. The MET proto-oncogene encodes the tyrosine kinase receptor for hepatocyte growth factor (HGF) and regulates various physiological processes including embryogenesis, wound healing, liver regeneration, angiogenesis, and immunomodulation [35]. In recent years, there has been interest in MET exon 14 alterations as potential drivers of lung cancer [42]. Exon 14 splice site alterations in MET have been identified as drivers of tumorigenesis [43]. However, the possibility of additional mechanisms controlling MET splicing, such as binding to other pre-mRNAs or interactions with other splicing factors, cannot be excluded and warrants further investigation.

AS events frequently occur in many tumors [5, 44, 45]. Until now, the role of hnRNPU in alternative splicing regulation in GC was unclear. Over the past decade, m^6^A modification has emerged as a crucial epigenetic mechanism for post-transcriptional regulation of gene expression. Aberrant m^6^A deposition has been implicated in GC progression and metastasis [46]. While multiple studies have elucidated the role of m^6^A in regulating alternative splicing [22, 47], the specific function of m^6^A in GC-associated AS events remains largely unexplored. In the present study, we characterize FTO-regulated AS events in GC cell lines and elucidate the complex interplay between m^6^A modification and AS, revealing both direct and indirect mechanisms of regulation. RIP and RNA pull-down assays demonstrated that FTO can bind to hnRNPU mRNA. Upon FTO knockdown, both the mRNA and protein levels of hnRNPU were dramatically decreased. Furthermore, we found that FTO specifically demethylates hnRNPU m^6^A modification within the 5' region. FTO knockdown also resulted in a decreased PSI of MET exon 14. These results suggest that FTO modulates MET exon 14 skipping indirectly via hnRNPU.

FTO, discovered as the first RNA m^6^A demethylase, is frequently dysregulated and plays significant roles in various types of cancers [24]. Elevated FTO expression has been reported in numerous tumors and represents an independent risk factor for poor overall survival in multiple cancer types [24]. FTO overexpression was initially identified in specific AML subtypes with characteristic genetic alterations. As an m^6^A demethylase, FTO removes m^6^A modifications from target mRNAs like ASB2 and RARA, promoting their degradation [18]. In non-small cell lung cancer, FTO has been demonstrated to enhance tumor proliferation through multiple m^6^A-dependent mechanisms, including activation of KRAS signaling and upregulation of USP7 and MZF1 [48]. Our study demonstrates that FTO is highly expressed in GC tissues, and elevated FTO expression correlates with poor prognosis. FTO knockdown inhibited GC cell growth in vitro and suppressed tumor growth in vivo. We corroborated these findings using the FTO inhibitor MA, which produced similar results to FTO knockdown. These findings suggest that FTO may serve as a potential therapeutic target for GC. Furthermore, FTO inhibitors could represent a promising avenue for targeted therapy in GC patients.

## Conclusions

This study demonstrates that aberrant alternative splicing plays a critical role in gastric cancer (GC) progression, with hnRNPU emerging as a key modulator of this process. Immunohistochemistry and Western blot analyses revealed hnRNPU overexpression in GC tissues and cells, which correlates with poor prognosis. Mechanistically, we found that the m6A demethylase FTO interacts with hnRNPU transcripts, reducing their m6A modification levels. RIP-seq and RNA-seq analyses further identified MET as a target gene of hnRNPU, with hnRNPU expression promoting exon 14 skipping of MET.

These findings elucidate a novel regulatory mechanism in GC progression involving the FTO-hnRNPU-MET axis. Our results suggest that targeting alternative splicing, particularly through modulation of hnRNPU or its associated pathways, may represent a promising therapeutic strategy for GC.

## Supplementary Information

Supplementary Material 1: Fig. S1. YTHDF3 is the m^6^A reader of hnRNPU. A. RIP assays to evaluate the interaction between hnRNPU and ALKBH5. The *p*-value was determined using an unpaired Student's t-test. B. qPCR to detect the mRNA of hnRNPU after overexpression FTO. The *p*-value was determined using an unpaired Student's t-test. C. Measurement of m^6^A levels after FTO overexpression. The *p*-value was determined using an unpaired Student's t-test. D. Analysis of hnRNPU mRNA decay rate. *p* value was calculated based on two-Way ANOVA.E-I. RIP assays to assess the binding between hnRNPU and IGF2BP1, IGF2BP2, YTHDF1, YTHDF2, or YTHDC2. The *p*-value was determined using an unpaired Student's t-test. J-M. Measurement of binding affinity between hnRNPU and YTHDF3 upon FTO knockdown or overexpression. The *p*-value was determined using an unpaired Student's t-test. Significant differences between groups are indicated as **p < 0.01 and ***p< 0.001. Fig. S2. FTO-hnRNPU axis play a crucial role in GC progression. A. Cell proliferation assessment using MTT assay following hnRNPU overexpression in FTO knockdown cells.* p* value was calculated based on two-Way ANOVA. B, D.Representative images of plate colony formation and soft agar assays. C, E. Quantification of colony numbers using ImageJ software. Data are presented as mean values ± standard deviation (SD) from triplicate experiments. *p* value was calculated based on one-Way ANOVA. Significant differences between groups are indicated as *p< 0.05 and **p < 0.01. Fig. S3. The AS of MET promote GC cell proliferation. A, B Examination of expression levels of MET-L (including exon 14) and MET-S (excluding exon 14) in GC cells. The *p*-value was determined using an unpaired Student's t-test. C, D. Cell proliferation assessment using MTT assay to evaluate the effects of MET-L and MET-S on GC cell growth.* p* value was calculated based on two-Way ANOVA. E, G. Representative images of plate colony formation and soft agar assays. F, H. Quantitative analysis of colony numbers using ImageJ software.* p* value was calculated based on one-Way ANOVA. I, J. Cell proliferation assessment by MTT assay following MET-L or MET-S overexpression in hnRNPU knockdown cells.* p* value was calculated based on two-Way ANOVA. Data are presented as mean values ± standard deviation (SD) from triplicate experiments. Significant differences between groups are indicated as *p < 0.05 and **p < 0.01.

## Data Availability

The authors declare that all data supporting the findings of this study are available in this article and its supplementary files.

## Abbreviations

AS: Alternative splicing
MA: Meclofenamic acid
GC: Gastric cancer
RBPs: RNA-binding proteins
FTO: Fat mass and obesity-associated protein
RIP: RNA immunoprecipitation
HGF: Hepatocyte growth factor
FBS: Fetal bovine serum
RT: Room temperature
CDX: Cell-derived xenograft
PDX: Patient-derived xenograft
MNU: N-methyl-N-nitrosourea
PCR: Polymerase chain reaction
IHC: Immunohistochemistry

## References

[1] Ajani JA, Lee J, Sano T, Janjigian YY, Fan D, Song S. Gastric adenocarcinoma. Nat Rev Dis Primers. 2017;3:17036. 28569272 10.1038/nrdp.2017.36

[2] Bray F, Laversanne M, Sung H, Ferlay J, Siegel RL, Soerjomataram I, et al. Global cancer statistics 2022: GLOBOCAN estimates of incidence and mortality worldwide for 36 cancers in 185 countries. CA Cancer J Clin. 2024;74(3):229–63. 38572751 10.3322/caac.21834

[3] Rawla P, Barsouk A. Epidemiology of GC: global trends, risk factors and prevention. Prz Gastroenterol. 2019;14(1):26–38. 30944675 10.5114/pg.2018.80001 PMC6444111

[4] Dicken BJ, Bigam DL, Cass C, Mackey JR, Joy AA, Hamilton SM. Gastric adenocarcinoma: review and considerations for future directions. Ann Surg. 2005;241(1):27–39. 15621988 10.1097/01.sla.0000149300.28588.23 PMC1356843

[5] Bonnal SC, Lopez-Oreja I, Valcarcel J. Roles and mechanisms of alternative splicing in cancer - implications for care. Nat Rev Clin Oncol. 2020;17(8):457–74. 32303702 10.1038/s41571-020-0350-x

[6] Wang GS, Cooper TA. Splicing in disease: disruption of the splicing code and the decoding machinery. Nat Rev Genet. 2007;8(10):749–61. 17726481 10.1038/nrg2164

[7] Bradley RK, Anczukow O. RNA splicing dysregulation and the hallmarks of cancer. Nat Rev Cancer. 2023;23(3):135–55. 36627445 10.1038/s41568-022-00541-7 PMC10132032

[8] Kahles A, Lehmann KV, Toussaint NC, Hüser M, Stark SG, Sachsenberg T, et al. Comprehensive analysis of alternative splicing across tumors from 8,705 patients. Cancer Cell. 2018;34(2):211-24.e6. 30078747 10.1016/j.ccell.2018.07.001 PMC9844097

[9] Dvinge H, Kim E, Abdel-Wahab O, Bradley RK. RNA splicing factors as oncoproteins and tumour suppressors. Nat Rev Cancer. 2016;16(7):413–30. 27282250 10.1038/nrc.2016.51 PMC5094465

[10] Bessa C, Matos P, Jordan P, Gonçalves V. Alternative splicing: expanding the landscape of cancer biomarkers and therapeutics. Int J Mol Sci. 2020;21(23):9032. 33261131 10.3390/ijms21239032 PMC7729450

[11] Shishkin SS, Kovalev LI, Pashintseva NV, Kovaleva MA, Lisitskaya K. Heterogeneous nuclear ribonucleoproteins involved in the functioning of telomeres in malignant cells. Int J Mol Sci. 2019;20(3):745. 30744200 10.3390/ijms20030745 PMC6387250

[12] Han BY, Liu Z, Hu X, Ling H. HNRNPU promotes the progression of triple-negative breast cancer via RNA transcription and alternative splicing mechanisms. Cell Death Dis. 2022;13(11):940. 36347834 10.1038/s41419-022-05376-6 PMC9643420

[13] Wang X, Xu J, Li Q, Zhang Y, Lin Z, Zhai X, et al. RNA-binding protein hnRNPU regulates multiple myeloma resistance to selinexor. Cancer Lett. 2024;580:216486. 37984724 10.1016/j.canlet.2023.216486

[14] Liang Y, Fan Y, Liu Y, Fan H. HNRNPU promotes the progression of hepatocellular carcinoma by enhancing CDK2 transcription. Exp Cell Res. 2021;409(1):112898. 34737140 10.1016/j.yexcr.2021.112898

[15] Huang H, Weng H, Chen J. m(6)A modification in coding and non-coding RNAs: roles and therapeutic implications in cancer. Cancer Cell. 2020;37(3):270–88. 32183948 10.1016/j.ccell.2020.02.004 PMC7141420

[16] Wang T, Kong S, Tao M, Ju S. The potential role of RNA N6-methyladenosine in cancer progression. Mol Cancer. 2020;19(1):88. 32398132 10.1186/s12943-020-01204-7 PMC7216508

[17] Zeng C, Huang W, Li Y, Weng H. Roles of METTL3 in cancer: mechanisms and therapeutic targeting. J Hematol Oncol. 2020;13(1):117. 32854717 10.1186/s13045-020-00951-w PMC7457244

[18] Li Z, Weng H, Su R, Weng X, Zuo Z, Li C, et al. FTO plays an oncogenic role in acute myeloid leukemia as a N(6)-Methyladenosine RNA Demethylase. Cancer Cell. 2017;31(1):127–41. 28017614 10.1016/j.ccell.2016.11.017 PMC5234852

[19] Hanniford D, Ulloa-Morales A, Karz A, Berzoti-Coelho MG, Moubarak RS, Sánchez-Sendra B, et al. Epigenetic silencing of CDR1as drives IGF2BP3-Mediated melanoma invasion and metastasis. Cancer Cell. 2020;37(1):55-70.e15. 31935372 10.1016/j.ccell.2019.12.007 PMC7184928

[20] Tsuchiya K, Yoshimura K, Inoue Y, Iwashita Y, Yamada H, Kawase A, et al. YTHDF1 and YTHDF2 are associated with better patient survival and an inflamed tumor-immune microenvironment in non-small-cell lung cancer. Oncoimmunology. 2021;10(1):1962656. 34408926 10.1080/2162402X.2021.1962656 PMC8366544

[21] Xiao W, Adhikari S, Dahal U, Chen YS, Hao YJ, Sun BF, et al. Nuclear m(6)A reader YTHDC1 regulates mRNA splicing. Mol Cell. 2016;61(4):507–19. 26876937 10.1016/j.molcel.2016.01.012

[22] Qiao Y, Sun Q, Chen X, He L, Wang D, Su R, et al. Nuclear m^6^A reader YTHDC1 promotes muscle stem cell activation/proliferation by regulating mRNA splicing and nuclear export. Elife. 2023;12:e82703. 36892464 10.7554/eLife.82703 PMC10089659

[23] Li S, Qi Y, Yu J, Hao Y, He B, Zhang M, et al. Nuclear Aurora kinase A switches m(6)A reader YTHDC1 to enhance an oncogenic RNA splicing of tumor suppressor RBM4. Signal Transduct Target Ther. 2022;7(1):97. 35361747 10.1038/s41392-022-00905-3 PMC8971511

[24] Li Y, Su R, Deng X, Chen Y, Chen J. FTO in cancer: functions, molecular mechanisms, and therapeutic implications. Trends Cancer. 2022;8(7):598–614. 35346615 10.1016/j.trecan.2022.02.010

[25] Deng X, Su R, Stanford S, Chen J. Critical enzymatic functions of FTO in obesity and cancer. Front Endocrinol (Lausanne). 2018;9:396. 30105001 10.3389/fendo.2018.00396 PMC6077364

[26] Relier S, Ripoll J, Guillorit H, Amalric A, Achour C, Boissière F, et al. FTO-mediated cytoplasmic m(6)A(m) demethylation adjusts stem-like properties in colorectal cancer cell. Nat Commun. 2021;12(1):1716. 33741917 10.1038/s41467-021-21758-4 PMC7979729

[27] Niu Y, Lin Z, Wan A, Chen H, Liang H, Sun L, et al. RNA N6-methyladenosine demethylase FTO promotes breast tumor progression through inhibiting BNIP3. Mol Cancer. 2019;18(1):46. 30922314 10.1186/s12943-019-1004-4 PMC6437932

[28] Huang J, Yang J, Zhang Y, Lu D, Dai Y. FTO promotes cervical cancer cell proliferation, colony formation, migration and invasion via the regulation of the BMP4/Hippo/YAP1/TAZ pathway. Exp Cell Res. 2023;427(1):113585. 37030332 10.1016/j.yexcr.2023.113585

[29] Yang S, Wei J, Cui YH, Park G, Shah P, Deng Y, et al. m(6)A mRNA demethylase FTO regulates melanoma tumorigenicity and response to anti-PD-1 blockade. Nat Commun. 2019;10(1):2782. 31239444 10.1038/s41467-019-10669-0 PMC6592937

[30] Zhang P, Xiang H, Peng Q, Ma L, Weng C, Liu G, et al. METTL14 attenuates cancer stemness by suppressing ATF5/WDR74/beta-catenin axis in GC. Cancer Sci. 2024;00:1–16. 10.1111/cas.16381 PMC11711053 39497511

[31] Fan HN, Chen ZY, Chen XY, Chen M, Yi YC, Zhu JS, et al. METTL14-mediated m(6)A modification of circORC5 suppresses GC progression by regulating miR-30c-2-3p/AKT1S1 axis. Mol Cancer. 2022;21(1):51. 35164771 10.1186/s12943-022-01521-z PMC8842906

[32] Singh S, Gupta S, Abhishek R, Sachan M. Regulation of m(6)A (N(6)-Methyladenosine) methylation modifiers in solid cancers. Funct Integr Genomics. 2024;24(6):193. 39438339 10.1007/s10142-024-01467-z

[33] Fang Z, Mei W, Qu C, Lu J, Shang L, Cao F, et al. Role of m^6^A writers, erasers and readers in cancer. Exp Hematol Oncol. 2022;11(1):45. 35945641 10.1186/s40164-022-00298-7 PMC9361621

[34] Jiang X, Liu B, Nie Z, Duan L, Xiong Q, Jin Z, et al. The role of m^6^A modification in the biological functions and diseases. Signal Transduct Target Ther. 2021;6(1):74. 33611339 10.1038/s41392-020-00450-x PMC7897327

[35] Recondo G, Che J, Janne PA, Awad MM. Targeting MET dysregulation in cancer. Cancer Discov. 2020;10(7):922–34. 32532746 10.1158/2159-8290.CD-19-1446 PMC7781009

[36] Graveel CR, Tolbert D, Vande Woude GF. MET: a critical player in tumorigenesis and therapeutic target. Cold Spring Harb Perspect Biol. 2013;5(7):a009209. 23818496 10.1101/cshperspect.a009209 PMC3685898

[37] Kim CH, Kim YD, Choi EK, Kim HR, Na BR, Im SH, et al. Nuclear speckle-related protein 70 binds to Serine/Arginine-rich splicing factors 1 and 2 via an Arginine/Serine-like region and counteracts their alternative splicing activity. J Biol Chem. 2016;291(12):6169–81. 26797131 10.1074/jbc.M115.689414 PMC4813587

[38] Cheong JH, Wang SC, Park S, Porembka MR, Christie AL, Kim H, et al. Development and validation of a prognostic and predictive 32-gene signature for GC. Nat Commun. 2022;13(1):774. 35140202 10.1038/s41467-022-28437-y PMC8828873

[39] Ule J, Blencowe BJ. Alternative splicing regulatory networks: functions, mechanisms, and evolution. Mol Cell. 2019;76(2):329–45. 31626751 10.1016/j.molcel.2019.09.017

[40] Shi ZD, Hao L, Han XX, Wu ZX, Pang K, Dong Y, et al. Targeting HNRNPU to overcome cisplatin resistance in bladder cancer. Mol Cancer. 2022;21(1):37. 35130920 10.1186/s12943-022-01517-9 PMC8819945

[41] Xiao R, Tang P, Yang B, Huang J, Zhou Y, Shao C, et al. Nuclear matrix factor hnRNP U/SAF-A exerts a global control of alternative splicing by regulating U2 snRNP maturation. Mol Cell. 2012;45(5):656–68. 22325991 10.1016/j.molcel.2012.01.009 PMC3299905

[42] Reungwetwattana T, Liang Y, Zhu V, Ou SI. The race to target MET exon 14 skipping alterations in non-small cell lung cancer: The why, the how, the who, the unknown, and the inevitable. Lung Cancer. 2017;103:27–37. 28024693 10.1016/j.lungcan.2016.11.011

[43] Frampton GM, Ali SM, Rosenzweig M, Chmielecki J, Lu X, Bauer TM, et al. Activation of MET via diverse exon 14 splicing alterations occurs in multiple tumor types and confers clinical sensitivity to MET inhibitors. Cancer Discov. 2015;5(8):850–9. 25971938 10.1158/2159-8290.CD-15-0285

[44] Sciarrillo R, Wojtuszkiewicz A, Assaraf YG, Jansen G, Kaspers GJL, Giovannetti E, et al. The role of alternative splicing in cancer: From oncogenesis to drug resistance. Drug Resist Updat. 2020;53:100728. 33070093 10.1016/j.drup.2020.100728

[45] Zhang Y, Qian J, Gu C, Yang Y. Alternative splicing and cancer: a systematic review. Signal Transduct Target Ther. 2021;6(1):78. 33623018 10.1038/s41392-021-00486-7 PMC7902610

[46] Li Y, Yuan Y. Alternative RNA splicing and GC. Mutat Res Rev Mutat Res. 2017;773:263–73. 28927534 10.1016/j.mrrev.2016.07.011

[47] Mendel M, Delaney K, Pandey RR, Chen KM, Wenda JM, Vågbø CB, et al. Splice site m(6)A methylation prevents binding of U2AF35 to inhibit RNA splicing. Cell. 2021;184(12):3125-42.e25. 33930289 10.1016/j.cell.2021.03.062 PMC8208822

[48] Li J, Han Y, Zhang H, Qian Z, Jia W, Gao Y, et al. The m^6^A demethylase FTO promotes the growth of lung cancer cells by regulating the m^6^A level of USP7 mRNA. Biochem Biophys Res Commun. 2019;512(3):479–85. 30905413 10.1016/j.bbrc.2019.03.093

