# Supplementary material for: hnRNPU-mediated pathogenic alternative splicing drives gastric cancer progression
Source: J Exp Clin Cancer Res. 2025 Jan 7;44:8. doi: 10.1186/s13046-024-03264-9 (PMC11705778; doi:10.1186/s13046-024-03264-9)

Alternatively splicing details of genes

1.Gene: ACTA2


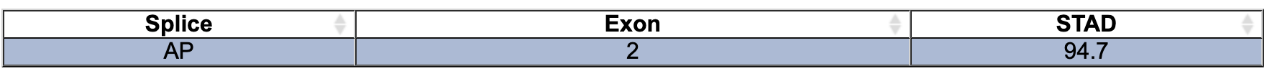


2.Gene: AREG


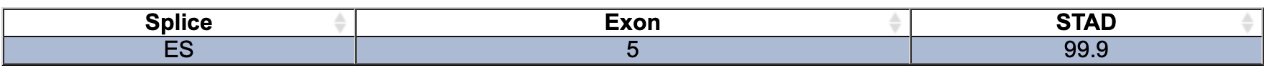


3.Gene:BRCA1
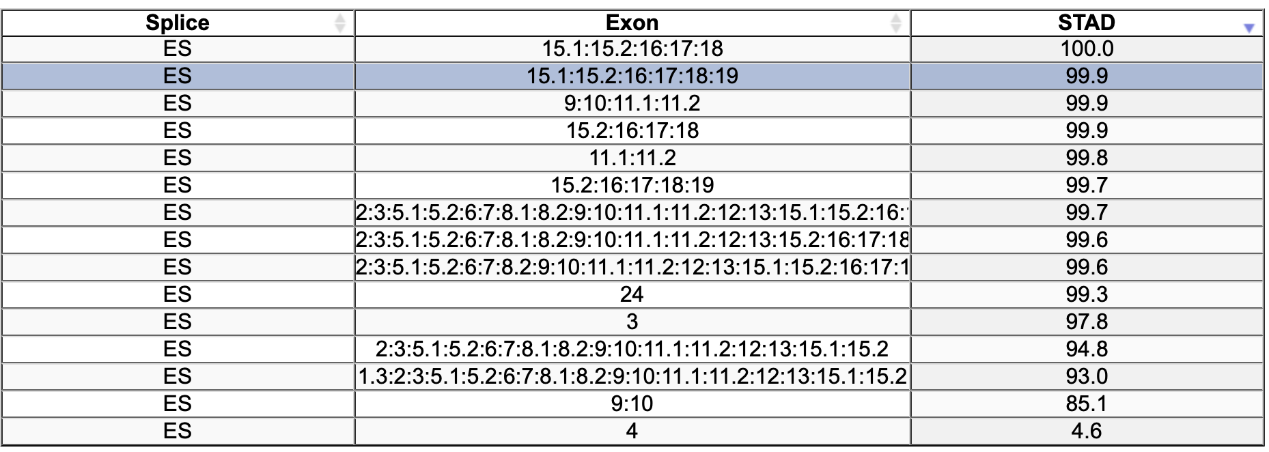


4.Gene: DDX5


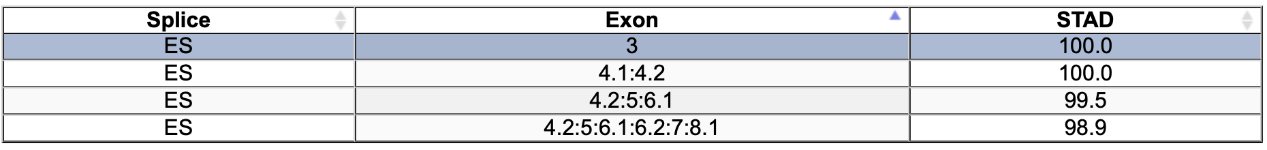


1. Gene: MSH6


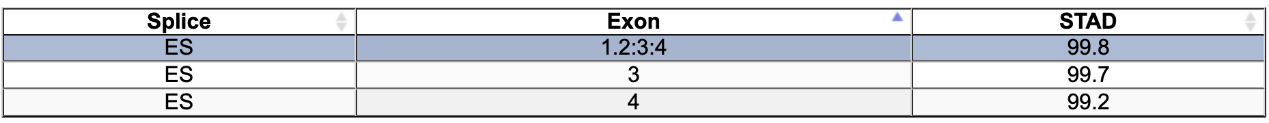


1. Gene: PAPR1


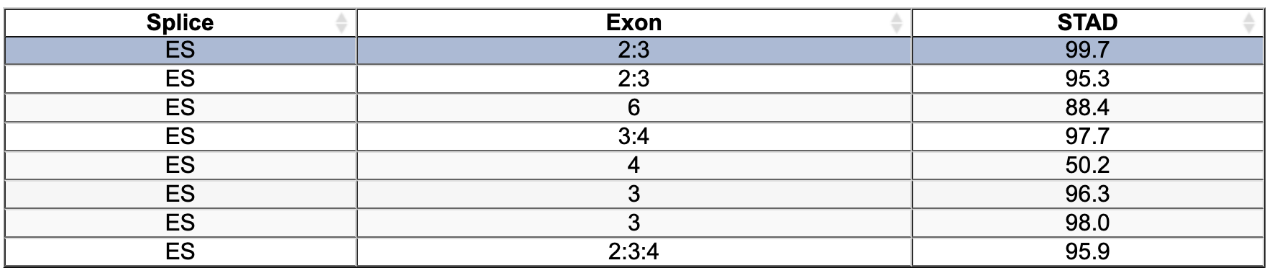

Supplement: Supplementary file 1 — Supplementary Material 1: Fig. S1. YTHDF3 is the m6A reader of hnRNPU. A. RIP assays to evaluate the interaction between hnRNPU and ALKBH5. The p-value was determined using an unpaired Student's t-test. B. qPCR to detect the mRNA of hnRNPU after overexpression FTO. The p-value was determined using an unpaired Student's t-test. C. Measurement of m6A levels after FTO overexpression. The p-value was determined using an unpaired Student's t-test. D. Analysis of hnRNPU mRNA decay rate. p value was calculated based on two-Way ANOVA.E-I. RIP assays to assess the binding between hnRNPU and IGF2BP1, IGF2BP2, YTHDF1, YTHDF2, or YTHDC2. The p-value was determined using an unpaired Student's t-test. J-M. Measurement of binding affinity between hnRNPU and YTHDF3 upon FTO knockdown or overexpression. The p-value was determined using an unpaired Student's t-test. Significant differences between groups are indicated as **p < 0.01 and ***p< 0.001. Fig. S2. FTO-hnRNPU axis play a crucial role in GC progression. A. Cell proliferation assessment using MTT assay following hnRNPU overexpression in FTO knockdown cells. p value was calculated based on two-Way ANOVA. B, D.Representative images of plate colony formation and soft agar assays. C, E. Quantification of colony numbers using ImageJ software. Data are presented as mean values ± standard deviation (SD) from triplicate experiments. p value was calculated based on one-Way ANOVA. Significant differences between groups are indicated as *p< 0.05 and **p < 0.01. Fig. S3. The AS of MET promote GC cell proliferation. A, B Examination of expression levels of MET-L (including exon 14) and MET-S (excluding exon 14) in GC cells. The p-value was determined using an unpaired Student's t-test. C, D. Cell proliferation assessment using MTT assay to evaluate the effects of MET-L and MET-S on GC cell growth. p value was calculated based on two-Way ANOVA. E, G. Representative images of plate colony formation and soft agar assays. F, H. Quantitative an [file 13046_2024_3264_MOESM1_ESM.docx]
